# Transporter-Mediated Drug Delivery

**DOI:** 10.3390/molecules28031151

**Published:** 2023-01-24

**Authors:** Gergely Gyimesi, Matthias A. Hediger

**Affiliations:** Membrane Transport Discovery Lab, Department of Nephrology and Hypertension, and Department for BioMedical Research, Inselspital, University of Bern, Freiburgstrasse 15, CH-3010 Bern, Switzerland

**Keywords:** membrane transporter, SLC, solute carrier, drug design, pharmacokinetics, prodrug, nanoparticle, bile acids

## Abstract

Transmembrane transport of small organic and inorganic molecules is one of the cornerstones of cellular metabolism. Among transmembrane transporters, solute carrier (SLC) proteins form the largest, albeit very diverse, superfamily with over 400 members. It was recognized early on that xenobiotics can directly interact with SLCs and that this interaction can fundamentally determine their efficacy, including bioavailability and intertissue distribution. Apart from the well-established prodrug strategy, the chemical ligation of transporter substrates to nanoparticles of various chemical compositions has recently been used as a means to enhance their targeting and absorption. In this review, we summarize efforts in drug design exploiting interactions with specific SLC transporters to optimize their therapeutic effects. Furthermore, we describe current and future challenges as well as new directions for the advanced development of therapeutics that target SLC transporters.

## 1. Introduction

In this review, we focus on the delivery of drugs via transmembrane transporters, especially those from the solute carrier (SLC) superfamily, through the cellular plasma membrane and various intracellular membranes. SLC solute carriers include all transmembrane proteins that enable the translocation of solutes such as nutrients, metabolites, ions and xenobiotics across the membrane in a facilitative or secondary active manner. Together with the ATP-dependent ABC (ATP-binding cassette) transporters (see below), transporter proteins form an essential protein machinery for the regulation of the cellular and systemic homeostasis of all solutes in our body, as well as for the maintenance of the necessary ion gradients, such as the inwardly directed Na^+^ gradient at the intestinal brush border membrane of enterocytes, to drive uptake of solutes in a Na^+^-coupled secondary active manner, as is the case for the intestinal uptake of glucose via SGLT1/SLC5A1 [1].

Transport proteins are often categorized by their mechanism of transport. ATP-binding cassette (ABC) transporters are primary active transporters that, in higher organisms, use ATP hydrolysis to drive the transport of solutes across the membrane and typically out of cells [2,3]. Secondary active transporters couple solute translocation to the cotransport or counter-transport of either inorganic ions or other solutes; in the latter case, they are often called exchangers. Facilitative transporters translocate solutes across the membrane according to their electrochemical gradient. As indicated earlier, secondary and facilitative transporters in both human and higher organisms are represented by the SLC solute carrier superfamily of proteins, a diverse, heterogenous group of proteins likely of polyphyletic origin [4,5].

The ABC and SLC transporter superfamilies in humans consist of 48 and over 400 proteins, respectively, out of which ~11 ABC transporters [6,7] and ~26 SLC transporters [6] are thought to be directly involved in drug translocation. Many of these transporters are present in the plasma membrane of liver and kidney cells, as well as in the cells of biological barriers, and thus profoundly shape the pharmacokinetics of small molecule drugs in the body [6]. Apart from exploring the interactions of existing drugs with transporters, approaches have been developed to exploit the unique cellular localization and transport activity of transporters as gateways for the delivery of therapeutics to specific organs and across specific biological barriers. In this review, we summarize such approaches and subsequently speculate on potential new directions and applications.

There are several factors that motivate the targeting of transporters expressed in specific tissues. For some medications, crossing certain barriers is vital for their action, e.g., central nervous system (CNS) drugs need to cross the blood–brain barrier (BBB). For others, it provides an advantage for therapeutic delivery, e.g., switching from intravenous to oral administration of chemotherapeutics across the intestinal barrier. In yet other cases, it can be beneficial for targeting drugs to specific cell types, e.g., targeting chemotherapeutics to tumor cells while sparing healthy cells. The unique and/or highly abundant expression of certain membrane transporters, such as bile acid transporters (SLC10A1/NTCP, SLC10A2/ASBT) and amino acid transporters (SLC7A5/LAT1, SLC6A14/ATB^0,+^), in specific cell types makes them good candidates for such efforts. Furthermore, unlike receptors, many transporters have a relatively promiscuous binding site for their ligands, making them more suitable for interaction with a broader range of small molecules [8], even though binding-site promiscuity can also lead to unwanted off-target effects [9]. Nevertheless, the substrates of transporters are usually small molecules that are stable, readily modifiable and non-immunogenic, whereas cell surface receptors are often tailored to interact with large molecules (e.g., low-density lipoprotein and transferrin) [8]. These aspects point to the particular advantage of using transporters instead of receptors for targeted drug delivery.

Knowledge of the distribution of transporter proteins in various human tissues is critical for understanding their role in drug metabolism and their usefulness as drug delivery targets. Because their importance in pharmacokinetics was recognized long ago, intensive studies have been conducted to determine the presence of transporters in the intestine, liver and kidney, the major organs that determine the ADMET (absorption, distribution, metabolism, excretion and toxicity) of drugs [6], as well as in the BBB, which determines the access of CNS drugs to the brain. For all oral formulations, the drugs must be able to cross the intestinal barrier after ingestion to be successfully absorbed into the body. The intestinal brush border membrane and mucus layer form the intestinal barrier [10], which is lined with numerous transporters for nutrient absorption [11,12]. After successful passage across the enterocytes, the absorbed drugs either enter the hepatic portal veins for direct delivery into the liver or, if they are lipophilic, enter the lymphatic system, thus avoiding the hepatic first-pass effect. The liver has a variety of promiscuous transporters capable of taking up a wide range of xenobiotics [6,11], whereupon they are metabolized by hepatocytes into less toxic and more water-soluble compounds [13]. The drug metabolites are then excreted back into the bloodstream or the bile via different transporters [13,14]. Polar drug metabolites are cleared from the body by the renal route, unless a specific transport mechanism exists in the renal proximal tubule cells for their reabsorption [15].

Certain organs (e.g., brain/CNS, retina and testes) are protected by a layer of endothelial cells that form barriers with regulated permeability through tight junctions between the blood and the underlying organ tissues. Drugs that act in these organs, particularly those that act in the CNS, must cross these barriers. It is well known that some of them utilize nutrient transporters present on the endothelial cell membranes [16,17] and it is estimated that 10–15% of all proteins in the neurovascular unit are membrane transporters [18]. In certain cases, it is necessary to prevent a drug that does not act in the CNS from reaching the brain. Therefore, knowledge of the presence of transporters at such barriers is of utmost importance, both for drug targeting and for avoiding undesirable off-target effects of drugs.

## 2. Strategies for Utilizing Transporters for Drug Delivery

In this section, we summarize several approaches to exploit transporters present in specific tissues for targeted drug delivery and more efficient drug absorption.

**Figure 1:**
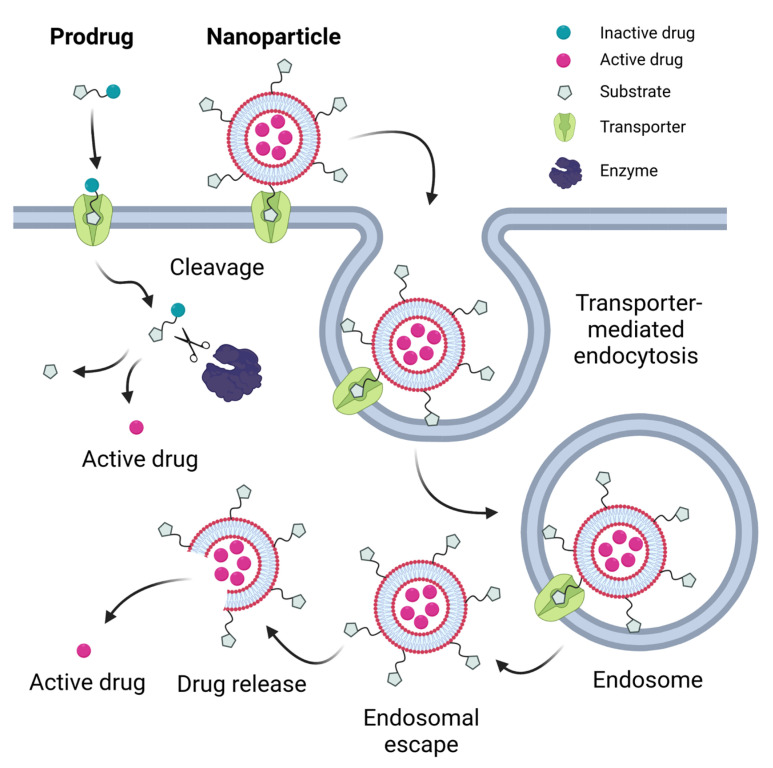
Illustration of the prodrug and functionalized nanoparticle strategies. In the prodrug strategy, the deliverable drug of interest is chemically linked to a known substrate of the target transporter. After uptake through the transporter, the chemical link is cleaved by intracellular enzymes, thus freeing the active drug molecule and an inert substrate. In the nanoparticle strategy, known substrates of the target transporter are chemically linked to the nanoparticle, which encapsulates the active drug molecules. Due to the size of the particle, binding to the transporter typically triggers endocytosis. Endosomal escape must occur after cellular uptake to release the content of the nanoparticles, the active drug, into the cytoplasm (see text). Created with BioRender.com.

A number of currently developed drugs have structural features in their molecular design that can be recognized by unique transporters expressed in specific tissues to improve their pharmacokinetic properties and minimize off-target effects [11]. A prodrug strategy is often followed, in which a known substrate molecule of an uptake transporter is chemically conjugated to the drug molecule of interest [12,19,20]. Recognition of the substrate moiety by the transporter then triggers uptake of the entire molecule into the cell. Once in the cytoplasm, the prodrug is designed to be cleaved and processed by active enzymes to release the active drug molecule (Figure 1). A review of prodrugs with targeted SLC-mediated absorption can be found in [12]. Numerous prodrug strategies have been developed targeting nutrient transporters to cross the intestinal or the blood–brain barrier. These transporters include transporters of amino acids (e.g., SLC7A5/LAT1), oligopeptides (SLC15A1/PEPT1), vitamins (SLC23A1/SVCT1, SLC5A6/SMVT), sugars (e.g., SLC5A1/SGLT1), bile acids (SLC10A2/ASBT) and carnitine (SLC22A5/OCTN2). Examples and recent developments of prodrugs utilizing these transporters are discussed in the next chapter.

It is often desirable that a drug is not transported into specific cells or across biological barriers to reduce off-target side effects. For example, first generation H1 histamine receptor antagonists, such as diphenhydramine, chlorpheniramine and cyproheptadine, are cationic drugs that exhibit sedative side-effects in the CNS because they can effectively enter the brain [21]. In contrast, second generation H1 antagonists, such as fexofenadine, cetirizine and ebastine, do not exhibit sedative side-effects. Fexofenadine and cetirizine have been shown to be substrates for ABCB1/P-gp, a key drug efflux transporter at the blood–brain interface, limiting drug availability in the brain [22,23,24]. Ebastine is rapidly converted to carebastine, which is also pharmaceutically active [25]. However, unlike ebastine, carebastine is also a good substrate for ABCB1/P-gp, while it is a poor substrate for the uptake transporters in brain capillary endothelial cells (BCECs), thereby likely contributing to reduced CNS side-effects [25]. These examples illustrate that, in certain cases, it may be advantageous to design drugs to be good substrates for drug efflux transporters to avoid their permeation across biological barriers.

A more recently developed and highly promising strategy for drug delivery is based on the production of nanoparticles, generally smaller than one micrometer, that can encapsulate drug molecules and release them under certain conditions. The purpose of encapsulation is either to increase the solubility of the drug molecule or to protect it from oxidizing conditions in the gastrointestinal tract. In this regard, it is important to note that there are physiological limitations to the size of the nanoparticles that can be used. Very small particles (<10 nm) are filtered in the renal glomerulus [26], while ~25 nm diameter is the enthalpic limit for the initiation of endocytic processes on the cellular surface according to kinetic models [27]. On the other hand, oversized particles (>200 nm) could activate the complement system, resulting in accumulation in the liver and spleen [28]. In order to enhance drug absorption and delivery, these nanoparticle scaffolds are often chemically modified. One such modification is the attachment of small molecule substrates of transporters to the surface of the nanoparticles to enhance recognition, and thus barrier passage or targeting to specific tissues (Figure 1).

A variety of chemical substances have been used as nanocarriers and substrates for further chemical modification to fine-tune pharmacokinetic properties. Among them, liposomes and liposome-based formulations (e.g., functionalized liposomes) [10,26,29,30,31], solid lipid nanoparticles/nanostructured lipid carriers [32], various polymer-based nanomicelles/nanoparticles [29,31,33,34,35,36,37,38,39,40], carbon dots [41], mesoporous silica nanoparticles [42,43] and nanoemulsions [44] have been used for transporter-targeted drug delivery.

Cellular uptake of such nanoparticles usually occurs through binding to the target transporter on the cell surface, followed by endocytosis via caveolin-dependent, clathrin-dependent or clathrin/caveolin-independent pathways such as micropinocytosis [10,26,29,30,44,45,46], rather than through uptake by the transporter itself (Figure 1). Endocytosed particles can be trapped in lysosomes, which can lead to their degradation and prevent their transcytosis, thus reducing their efficiency [47,48]. After the endocytic process, transporters are usually restored/recycled, but the exact mechanism remains unclear [26].

In the next chapter we review transporters that have been used as targets of either the prodrug approach or to nanoparticle targeting.

## 3. Targeting Transporters

### 3.1. Facilitative Glucose Transporters (GLUTs)

Facilitative glucose transporters are members of the GLUT/SLC2 family, which includes 14 transporters in humans [49]. Most members of this family transport sugars with a six-membered ring, such as glucose and galactose, with different substrate specificities and tissue expression patterns depending on their biological roles. The best-characterized member, GLUT1/SLC2A1, is present to varying degrees in many different tissues and cell types. Particularly high expression levels are found in erythrocytes, endothelial cells of the blood–brain barrier (BBB) and endometrial stromal cells of the placenta, where GLUT1 fulfills vital physiological roles [49]. At the BBB, for example, GLUT1 is the major mechanism for glucose delivery to the central nervous system (CNS), and mutations in its SLC2A1 gene cause GLUT1 deficiency syndrome with seizures and other neurological symptoms [50]. Other members of the GLUT/SLC2 family have more tissue-specific expression patterns, such as GLUT5/SLC2A5 and GLUT7/SLC2A7, which are abundant in the small intestine, and GLUT4/SLC2A4, which represents the major glucose uptake pathway in skeletal muscle [49]. GLUT1 has attracted particular attention as its expression has been detected both at the blood–brain barrier and in malignant glioma cells that have elevated nutrient demand [51].

The prodrug strategy was used early on with GLUT1 to deliver drugs to the CNS. D-glucose and D-galactose esters of 7-chlorokynurenic acid, an *N*-methyl-D-aspartate (NMDA) receptor antagonist, were synthesized as prodrugs to facilitate the delivery of 7-chlorokynurenic acid to the CNS and were shown to be effective against NMDA-induced seizures in mice [52,53]. Glycosyl derivatives of dopamine and L-DOPA (L-3,4-dihydroxyphenylalanine), synthesized as anti-Parkinson prodrugs, were also shown to be effective in classic dopaminergic models, indicating that the prodrugs can cross the BBB and act in the CNS after intravenous administration [54]. In later in vitro studies, a glucose-dopamine conjugate was shown to compete with 3-*O*-methylglucose, a non-metabolizable substrate of GLUT1, indicating direct involvement of the transporter in the uptake process [55]. Interestingly, the conjugation of chlorambucil, an anticancer drug, with glucose yielded a compound that interacts with GLUT1 but is not itself transported [56].

GLUT1 has been used to deliver diagnostic and imaging markers into tumor cells; for example, [^18^F]fluoro-2-deoxy-D-glucose is used in positron emission tomography for in vivo tumor diagnosis [57,58]. Similarly, γ-Fe_2_O_3_ nanoparticles were coated with dimercaptosuccinic acid and modified with 2-deoxy-D-glucose to target GLUT1-overexpressing cells for tumor imaging [59]. Liposomes loaded with the fluorescent dye coumarin 6 and decorated with glucose residues bound to cholesterol via poly(ethylene glycol) (PEG) also successfully delivered coumarin 6 to mouse brain [60]. Glucose-functionalized poly(lactic-*co*-glycolic acid) (PLGA) nanoparticles were also developed and shown to deliver the encapsulated Cy5.5 fluorescent dye into HEp-2 cells that express GLUT1 at high levels, and enhanced antiproliferative effects were demonstrated when these nanoparticles were loaded with the chemotherapeutic agent docetaxel [37].

In addition, the GLUT1 pathway has been exploited to target gliomas, requiring therapeutics to both cross the BBB and achieve maximal distribution in the tumor tissue. GLUT1 is a promising candidate because it is expressed in both the BBB and various tumors. For glioma therapy, 2-deoxy-D-glucose-functionalized poly(ethylene glycol)-*co*-poly(trimethylene carbonate) (PEG-PTMC) nanoparticles were used to effectively enhance binding to GLUT1 in a dual targeting strategy involving both BBB transfer and tumor penetration [36]. Specifically, PEG-PTMC, a biodegradable aliphatic polycarbonate, was conjugated with 2-deoxy-D-glucose to target GLUT1-mediated transcytosis and deliver the encapsulated anticancer drug paclitaxel to the brain. The 2-deoxy-D-glucose nanoparticles were shown to be effectively internalized by the cells through caveolae- and clathrin-mediated endocytosis [36]. In addition, both an in vitro co-culture model of the BBB and in vivo studies with mice showed effective uptake and anti-glioma activity when the nanoparticles were loaded with paclitaxel [36]. A similar technique was used with D-glucosamine-functionalized nanoparticles, which showed enhanced tumor uptake and antiproliferative activity in cancer cells, 3D tumor spheroids and in vivo mouse xenografts [61]. Due to the high affinity of GLUT1 for D-glucosamine, these nanoparticles could enter the tumor tissue through GLUT1-mediated endocytosis with improved selectivity.

Functionalized nanoscale particles decorated with dehydroascorbic acid (DHA) have also been used to target GLUT1 for drug delivery, e.g., across the BBB or into malignant gliomas [62,63]. GLUT1, which is expressed on endothelial cells of the BBB, can transport not only glucose but also DHA into the brain, which is subsequently reduced to ascorbic acid [64]. Because ascorbic acid is not a substrate for GLUT1, DHA transport is unidirectional, making this system ideal for drug delivery via the BBB. In one study, a “smart nanodevice” was built and decorated with DHA using click chemistry to target malignant glioma cells [63]. The nanodevice was loaded with paclitaxel via disulfide bonds, which protects the entrapped drug from escaping into the bloodstream but are reduced inside the cell due to the high concentration of glutathione, triggering the release of the drug. These nanoparticles showed significantly enhanced targeting to glioma and enhanced chemotherapeutic effects. A similar strategy was used to deliver itraconazole into the brain as a therapy against intracranial fungal infection. Compared with the non-conjugated micellar formulations, this strategy showed a significantly higher efficacy [62].

Multivalent glucosides have also been used as ligands to functionalize liposomes for enhanced brain delivery by targeting GLUT1 [65]. The modified nanoparticles were able to deliver docetaxel into the brains of mice with significantly higher efficiency than unmodified liposomes or the direct application of docetaxel alone. Modification with quinantennary glucoside yielded the highest efficiency of delivery into the brain [65].

GLUT1 has been shown to be overexpressed not only in gliomas, but also in other cancers outside the CNS as well. One example is hepatocarcinoma, against which a micellar formulation of PEG-*p*Lys-*p*Phe polymers decorated with dehydroascorbic acid was developed, again anchoring the drug via a disulfide link, the dissociation of which triggers release of the encapsulated drug due to high intracellular glutathione levels [38]. Such doxorubicin-loaded nanocarriers showed remarkable targeting abilities to hepatocarcinoma cells and enhanced anti-tumor efficacy [38]. Mesenchyme-like cancer cells were furthermore targeted by glucose-coated magnetic nanoparticles, with glucose shown to compete with nanoparticle uptake, suggesting the direct involvement of sugar transporters in the uptake process [66].

Because GLUT1 is abundantly expressed in the BBB, it has been targeted for drug delivery for the treatment of neurodegenerative diseases, as discussed in the following examples.

Glucose-decorated nanomicelles were engineered for brain delivery of 3D6 antibody fragments (3D6-Fab) used for the clearance and reduction of Aβ plaques in Alzheimer’s disease, where glucose decoration was responsible for a marked increase in cellular uptake [35]. Uptake was inhibited in a dose-dependent manner by the GLUT1 inhibitor phloretin, indicating the involvement of GLUT1 in the process [35]. The highest level of brain penetration measured in mice was achieved with a 25% glucose decoration ratio, while the enhancement of Fab uptake into peripheral tissues was negligible. The delivered 3D6-Fab was also successful in preventing the aggregation of Aβ in a mouse model of Alzheimer’s disease [35]. Proper orientation of the glucose molecule on the nanomicelle surface (i.e., conjugation through the C_6_ position of glucose) was found to be crucial for glucose-GLUT1 interactions and nanoparticle entry into the brain [67].

A PEG-based polymeric formulation was conjugated with galactose to enhance brain delivery of anti-BACE1 siRNA against Alzheimer’s disease, based on the observation that D-glucose and D-galactose are both substrates of GLUT1 [68]. The galactose-modified nanoparticles showed cellular uptake that was inhibited by phloretin in a dose-dependent manner, indicating a dominantly GLUT1-mediated uptake pathway [69], while their brain penetration was 5.8-fold higher than that of nanoparticles not modified with galactose [69]. The effect of galactose-mediated targeting was underscored by behavioral studies in the APP/PS1 double transgenic mouse model of Alzheimer’s disease, which showed that in contrast to mice treated with non-galactose-modified nanoparticles, mice treated with the galactose-decorated anti-BACE1 siRNA-loaded nanoparticles achieved the performance of normal, healthy WT mice in the novel object recognition test [69].

It is important to emphasize that conjugation with a ligand transported by a specific transporter does not automatically mean that that transporter is the primary uptake route. Glucose-coated nanoparticles have been shown to cross the primary human brain endothelium at least three times faster than non-brain endothelia, with eventual localization in astrocytes [70]. However, the GLUT1 inhibitor cytochalasin-B had no effect on the rate of transport of these molecules. It was first assumed that uptake occurs through passive diffusion, as vesicular transport could not be detected, but uptake and transfer rates are temperature dependent, suggesting that other cellular processes are involved.

The high or specific expression of other glucose transporters such as GLUT2/SLC2A2, GLUT3/SLC2A3, GLUT12/SLC2A12 and the fructose transporter GLUT5/SLC2A5 has also been associated with various cancer types and specific cancer stages [71,72,73,74]. For example, GLUT5 shows a 5-fold and 17-fold higher protein expression in MCF-7 and MDA-MB-231 breast cancer cell lines, respectively, compared to the 184B5 non-cancerous breast cell line [75]. However, the lack of specific binders to these transporters has hindered the development of therapeutics that can utilize these proteins [75]. In the case of GLUT5, fluorescently labeled glycoconjugates (2,5-anhydro-D-mannitol-coumarines) have shown high affinity and specificity of binding [76,77]. Based on these results, either mannitol directly, or mannitol–coumarin were chemically conjugated with chlorambucil, an anticancer agent [75]. These prodrugs showed selective uptake into the GLUT5-expressing MCF-7 breast cancer cell line compared with the 184B5 non-cancerous mammary tissue cell line, which competed with the uptake of previously used fluorescent probes, showing a GLUT5-dependent uptake mechanism. All but one of the synthesized prodrugs also showed a cytotoxic effect [75].

Attempts were also made to target GLUT4/SLC2A4 in muscle cells. GLUT4 in the C1C12 muscle cell line was targeted with glucose-functionalized quantum dots, and uptake responded to insulin stimulation, which is known to increase the surface expression of GLUT4, and competed with 2-deoxyglucose, suggesting the direct involvement of GLUT4 in the uptake process [78].

### 3.2. Amino Acid Transporters

LAT1/SLC7A5 has been in the spotlight of drug delivery efforts because it is abundant in BCECs of the BBB as well as in glioma cells and other tumors [79,80,81]. For this reason, there has been a great focus on LAT1-mediated drug delivery, either with the goal of delivering therapeutics to the CNS or to specifically target cancer cells. It has been argued that LAT1 has better properties than 20 other transporters studied for delivery across the BBB in terms of high maximal capacity and appreciable binding affinity, relatively simple structural requirements for binding and relative promiscuity, and the fact that neither its use nor the disruption of its activity by the possible overdose of therapeutics result in irreversible brain damage [82,83,84]. LAT1 is prominently expressed on both the luminal and abluminal sides of the BBB [80] and its expression was not altered by inflammatory insult in the mouse BBB [85]. LAT1 is a non-glycosylated protein [86,87] that forms an obligate complex with the *N*-glycosylated auxiliary type II membrane protein 4F2hc/SLC3A2, resulting in a transport system that is also referred to as system L [86,88].

Several drugs already utilize LAT1 for crossing the BBB (e.g., melphalan [89], levodopa [79], gabapentin [90], pregabalin [91], methyldopa and baclofen [26]). The approach to generating LAT1-transported prodrugs mostly utilizes the conjugation of drugs with large and/or hydrophobic amino acids such as L-Phe and L-Tyr, and has been applied to drugs such as ketoprofen [92], ferulic acid [83], dopamine [93], valproic acid [94], nipecotic acid [95], phosphonoformate [96], flurbiprofen, salicylic acid, ibuprofen, naproxen [87] and probenecid [97] (for a recent review, see [98]). In addition, gemcitabine has also been conjugated with threonine to target LAT1 [99]. There is also a prodrug strategy to enable the brain penetration of 7-chlorokynurenic acid, an NMDA receptor antagonist, by converting it into the prodrug of 4-chlorokynurenine [100]. This compound is an amino acid that is readily taken up by the system L (LAT1/SLC7A5) through the BBB into the CNS [100]. In terms of substrate recognition by LAT1, analysis of competent substrates has suggested that a free amino and a free carboxyl group are required for recognition by LAT1 [101,102,103]. A pharmacophore study later found that the free amino group can also interact with LAT1 through a H-bond interaction instead of purely through the positive charge [104]. The model has also pointed out the preference for aromatic vs. lipophilic moieties, as well as an optional H-bond acceptor region that can enhance affinity [104]. Later, a quantitative structure-activity relationship (QSAR) model was developed for designing potent binders of LAT1, which indicated that meta-substituted amide derivatives of phenylalanine (i.e., with an amide bond at the meta-position of the aromatic ring) have the highest ability to utilize LAT1 [105]. Subsequently, a detailed in vitro study was performed on the structural features affecting the transportability of LAT1-targeted phenylalanine-drug conjugates, providing further insight into suitable drug scaffolds for selective and efficient delivery via this strategy [106]. It should be noted that prodrugs designed to be substrates for LAT1 can also be substrates for other uptake transporters, such as monocarboxylate transporters 8 and 10 (MCT8/SLC16A2 and MCT10/SLC16A10, respectively), and organic anion transporter proteins (OATPs, SLCO/SLC21 family) [107,108]. Eadie–Hofstee plots can be applied to find uptake systems that transport a particular compound, and such additional transport systems have been found for a number of prodrugs at higher concentrations [87].

Nanoparticles have also been functionalized using LAT1 substrates to focus their targeting. In particular, LAT1/SLC7A5 has been used to deliver anticancer medication through the BBB for the treatment of glioma, either for imaging/detection/staging purposes or for developing anti-cancer therapeutics.

Gold nanoclusters (AuNCs) have recently emerged not only as a promising detection approach in biomedical imaging, but also in drug delivery by conjugating drugs to the AuNCs [109]. For the delivery of doxorubicin into cancer cells, methionine and the fluorescent dye MPA were conjugated with AuNCs and doxorubicin was immobilized on the methionine-modified AuNCs to form Au-Met-DOX nanoparticles [110]. The authors propose that LAT1/SLC7A5 and LAT2/SLC7A8, which transport methionine into malignant cells, are involved in the drug delivery process [110].

The functionalized nanoparticle strategy was also used to target LAT1 by L-DOPA-decorated amphiphiles [111]. These liposomes, when loaded with NIR-dye, showed preferential accumulation in brain tissue, and while carrying WP1066, a STAT3 inhibitor [112], enhanced overall survival in a glioblastoma mouse model [111].

An interesting approach is presented by Mintz et al., who synthesized carbon nanodots from tryptophan and 1,2-ethylenediamine, which were able to cross the BBB in zebrafish [41]. The authors hypothesized that residual tryptophan bound to the surface of the carbon dots facilitated uptake through the BBB via the LAT1 transporter [41].

Phenylalanine-conjugated solid lipid nanoparticles were prepared that can deliver doxorubicin into glioma with higher efficiency than without phenylalanine conjugation [113]. However, these efforts have been criticized because the conjugation was performed on the α-carboxyl group of phenylalanine, which was previously reported to be essential for recognition by LAT1 [104,114]. Moreover, phenylalanine tends to be entrapped in the core of solid lipid nanoparticles due to its hydrophobicity [103].

Later on, a revised strategy for the delivery of doxorubicin into glioma was utilized, involving the conjugation of the γ-carboxyl group of glutamate to the surface of liposomes and PLGA nanoparticles, leaving the α-amino and α-carboxyl groups free for recognition by LAT1. This approach resulted in effective transcytosis across the BBB and uptake into glioma cells [103]. A similar approach was applied to PLGA nanoparticles to target breast cancer cells through LAT1-mediated delivery [115].

Liposomes composed of egg phosphatidylcholine (EPC) and dioleoyl phosphatidylethanolamine (DOPE) and modified with an L-tyrosine conjugated polymer showed enhanced uptake into HeLa cells, which strongly express LAT1, compared to liposomes with unconjugated polymer [116]. Interestingly, the polymer used, poly(*N*-isopropylacrylamide-*co*-*N*,*N*-dimethylacrylamide), is a thermoresponsive polymer that exhibits a phase transition at 32 °C, leading to changes in hydrophobicity associated with its hydration and dehydration. At temperatures above the phase transition temperature, the uptake of the formulated nanoparticles into HeLa cells was observed because the polymer surface of the liposomes became hydrophobic [116]. L-tyrosine modification further enhanced the cellular uptake of these nanoparticles [116].

In addition to LAT1, another commonly targeted amino acid transporter is SLC6A14/ATB^0,+^. SLC6A14 is a relatively promiscuous transporter that transports a wide range of neutral and cationic amino acids [117] and is highly concentrative [8]. It is prominently expressed in the lungs [117] but is also found in the small intestine [118,119], making it a candidate for oral drug delivery. In addition, SLC6A14 has been shown to be upregulated in various tumor types [120,121,122,123].

SLC6A14 has been targeted by prodrugs as it can transport a wide range of substrates, conjugation with an amino acid by esterification and acylation transforms many drugs into substrates of SLC6A14 [124]. Examples include valacyclovir (the L-valyl ester of the antiviral drugs acyclovir), valganciclovir (the L-valyl ester of the antiviral drug ganciclovir), the L-glutamic acid γ-ester of acyclovir [124] and Val-SN-38, the valyl ester prodrug of the topoisomerase inhibitor SN-38, itself a derivative of irinotecan [125]. Interestingly, Val-SN-38 was also taken up by SLC38A1, SLC38A2 and ASCT2/SLC1A5, highlighting that several transporters with overlapping substrate specificity can contribute to prodrug uptake [125]. The quaternary ammonium substrate of SLC6A14, L-carnitine, has also been used as a vehicle for the delivery of small molecules, such as butyrate, in a prodrug approach. Butyrate is a bacterial metabolite that has been attributed with preventive effects against inflammation in the large intestines, as well as tumor suppression and immunosuppressive effects relevant to the treatment of inflammatory bowel disease (IBD) [126]. Since SLC6A14 has been shown to be upregulated under IBD conditions [126], an ester prodrug of butyrate conjugated with L-carnitine was developed to target colon epithelial cells [126]. The butyrate-L-carnitine prodrug has been shown to interact with both SLC6A14 and the intestinal carnitine transporter OCTN2/SLC22A5 [126] (see also the section about organic cation transporters).

In a study using liposomes conjugated with small molecules, different amino acids (Gly, Asp, Lys) were evaluated for their targeting efficacy, with lysine showing the highest efficacy [30,127]. Lysine-conjugated liposomes, which were proposed to directly interact with SLC6A14, have been shown to be taken up by carcinoma cell lines [30]. The authors suggest that the binding of the lysine-conjugated particle leads to a sustained occluded state of the transporter, which induces endocytosis [30]. The preparation further showed selective accumulation of lysine-conjugated liposomes in tumor cells compared to non-conjugated liposomes. Aspartate conjugates also showed efficacy when the conjugation occurred via the β-carboxyl group of the aspartate side chain.

In addition, L-valine-conjugated PLGA nanoparticles have been used to improve the oral delivery of insulin, presumably through uptake by amino acid transporters in the small intestine [128]. However, in this case, the authors mention that L-valine was conjugated with the PLGA particles through its amino group, which could preclude their binding to amino acid transporters.

Attempts were also made to target both LAT1 and SLC6A14 with a single nanoparticle [129]. Liposomes loaded with the anticancer agent irinotecan, the water-soluble precursor of its active metabolite SN-38, were functionalized with polyethylene glycol monostearate conjugated with different amino acids. Interestingly, while liposomes functionalized with glutamate and lysine were able to target LAT1 and SLC6A14, respectively, tyrosine-functionalized liposomes were able to target both transporters simultaneously. These dual-targeting liposomes showed highest uptake efficiency in BxPC-3 and MCF-7 cancer cells, which highly express both LAT1 and SLC6A14. The tumor inhibition rate of the tyrosine-functionalized liposomes was also higher than that of unfunctionalized liposome formulations. The mechanism of uptake was confirmed as LAT1- and SLC6A14-mediated endocytosis [129].

Another amino acid transporter, ASCT2/SLC1A5, has been shown to be overexpressed in various carcinomas, making it an ideal target for cancer-specific drug delivery [130,131,132,133,134]. ASCT2 is a sodium-dependent neutral amino acid exchanger that transports L-alanine and L-glutamine as well as other small neutral amino acids [135,136,137,138]. Glutamine transport activity is particularly important for cancer cells, in which glutamine addiction can develop due to the Warburg effect [139]. For this reason, radiolabeled [^18^F](2*S*,4*R*)4-fluoroglutamine has been used as a positron emission tomography (PET) probe for tumor imaging [140,141]. Similarly, non-glutamate based amino acid radiotracers have been developed for use with PET/CT-based cancer diagnosis, such as anti-1-amino-3-[^18^F]fluorocyclobutane-1-carboxylic acid (FACBC, fluciclovine) [142,143], which is also partly transported by ASCT2 [144]. The prodrug strategy has additionally been used to generate a series of glutamine-linked platinum (IV) prodrugs that have shown anticancer activity, albeit to a lesser extent than their parent compound, cisplatin. However, for one of the compounds, the authors expect less off-target accumulation, as it is mainly taken up by ASCT2-overexpressing tumor cells [145].

Functionalized nanoparticles targeting ASCT2 via glutamine have also been generated. In one study, glutamine-β-cyclodextrin was synthesized and loaded with doxorubicin. It was shown to specifically accumulate in strongly ASCT2-expressing triple negative breast cancer cell lines (MDA-MB-231 and BT549). Uptake of the nanoparticles was attenuated by L-γ-glutamyl-*p*-nitroanilide, a specific inhibitor of ASCT2, demonstrating the involvement of the transporter in the uptake process [146]. In another study, a polyglutamine-based co-polymer gene delivery system was developed for cancer therapy to deliver interfering siRNA agents against multidrug resistance protein 1 (MDR1/P-gp/ABCB1) and survivin. The nanoparticles were shown to be taken up by clathrin-mediated endocytosis, which was partially ASCT2-dependent, as the inhibition of ASCT2 attenuated uptake, while glutamine deprivation enhanced it [147]. Interestingly, nanoparticle binding resulted in a significant decrease in intracellular glutamine levels due to competition for glutamine, which in turn resulted in a remarkable upregulation of ASCT2. In vivo, the polyglutamine-based nanoparticles were shown to be specifically taken up into the lung parenchyma after intravenous injection, likely due to the high expression of ASCT2 in that tissue [147].

As mentioned earlier, LAT1 and also other SLC7 amino acid transporters form obligate complexes with type II single-transmembrane domain glycoproteins of the SLC3 family [133,148,149,150]. Thus, SLC3A2/4F2hc (also known as CD98), the obligate interaction partner of LAT1, although not a transporter itself, is a potential target as well due to its elevated expression in various carcinomas and neoplasms, and as a consequence of intestinal inflammation [151,152].

Targeting and silencing of SLC3A2 in colorectal cancer was the basis for the development of a new oral nanoparticle strategy that improves the efficacy of anticancer drugs. While SLC3A2 is only weakly expressed on the basolateral membranes in healthy colon epithelial cells, it is distinctly overexpressed on both apical and basolateral membranes in colon cancer, where it plays a special role in the development of colon cancer [153]. This suggests that SLC3A2 can serve as a receptor for targeted drug delivery in colon cancer cells and that its downregulation, in combination with anticancer drug treatment, increases the therapeutic efficacy of the anticancer drug. For this purpose, SLC3A2 siRNA and the anti-cancer alkaloid camptothecin were co-loaded into SLC3A2 Fab-functionalized nanoparticles [153]. These nanoparticles showed enhanced drug delivery, anticancer and antimigration effects in in vitro and in vivo experiments compared with drug-only loaded nanoparticles or non-functionalized nanoparticles, demonstrating the potential of this targeted nanoparticle combination therapy [153]. A similar antibody-mediated targeting strategy was used with PLGA nanoparticles to deliver SLC3A2 siRNA into intestinal cells of mice with colitis [154], thereby targeting SLC3A2 on the surface of colon epithelial cells and macrophages, where it is overexpressed due to inflammatory processes [152,154].

### 3.3. Bile Acid Transporters

Bile acid conjugation was used as early as 1948 as a strategy for targeting hepatocytes to treat germ and viral infections attacking the liver [10,155,156]. Bile acids are polyhydroxylated steroidal acids derived from cholesterol that are secreted by the liver into the bile canaliculus via the ABCB11/BSEP transporter and stored in the gallbladder [157]. Most of the chemical species in bile are the primary bile acids cholic acid and chenodeoxycholic acid, which are conjugated with either glycine or taurine [157]. After emptying into the small intestine following the ingestion of a meal, they help solubilize and break down large dietary lipid droplets by converting them into small ones, thus improving accessibility to pancreatic lipases. After enzymatic digestion of the components of the micelles, the lipids are converted into common hydrolysis products such as fatty acids, monoacylglycerols, phospholipids and free cholesterol [10,158]. These products remain associated with the bile acids in mixed micelles, which facilitate their passage through the intestinal mucus layer, one of the important barriers to overcome when developing drug delivery strategies [10,159]. The ingredients of the micelles are then taken up by the enterocytes in the upper part of the small intestine, either via passive diffusion or by specific transporter proteins [10,160,161,162]. After uptake across the brush border membrane, the resulting intracellular lipid droplets bind to fatty acid binding protein (FABP), and the lipid digestion products migrate to the endoplasmic reticulum, where they are re-esterified to generate triglycerides, phospholipids, cholesterol esters, etc. After transport from the ER to the Golgi apparatus, lipids are packaged together with apolipoproteins to form chylomicrons, with apolipoproteins playing an important role in chylomicron synthesis [163,164]. The chylomicrons are then extruded from the Golgi apparatus in exocytic vesicles and released across the basolateral membrane into the lacteals in the villi of the small intestine, and thereafter into the lymphatic vessels and thoracic duct to enter the systemic circulation [10,160,161]. Similar transport pathways exist for certain fat-soluble vitamins, carotenoids and other lipophilic compounds that rely on the formation of bile acid micelles.

As for the absorption of therapeutic drugs via the lipid absorption pathway, most of them enter the portal vein after transcytosis by enterocytes. However, it is also known that the bioavailability of several highly lipophilic drugs depends significantly on lymphatic transport [161,165,166]. In general, it is the high lipophilicity and the large particle size that favor the lymphatic system over the portal vein [10]. Drug uptake via the lymph has several advantages, such as the ability to bypass first-pass metabolism in the liver and the avoidance of rapid distribution of drugs into organs and tissues, thus resulting in reduced toxicity [10,161,165].

The lymphatic delivery pathway can also be exploited via the microfold M cells located in the intestinal epithelium [166]. These are specialized immune cells distributed among the epithelial cells covering mucosa-associated lymphoid tissues such as Peyer’s patches [10]. Their normal function is to rapidly take up antigens from the intestinal lumen in order to initiate an immune response [10,167,168]. They lack microvilli and a mucus layer and are coated with a thinner glycocalyx than enterocytes, which allows them easier access to the contents of the intestinal lumen, making them ideal candidates for developing advanced oral bioavailability strategies for therapeutics [10,167].

While lipid absorption facilitated by bile acid micelles occurs in the upper part of the small intestine, conjugated bile acids are taken up via the luminal sodium-coupled bile acid transporter SLC10A2/ASBT located in the distal ileum. There, 95% of conjugated bile acids are absorbed as part of the recycling of bile salt called enterohepatic circulation, which is important because the liver is unable to synthesize enough bile salts to meet the daily requirements [10,169]. As part of the recycling process, bile acids taken up through the apical membrane of epithelial cells via SLC10A2/ASBT bind to the ileal bile acid binding protein IBABP, which then shuttles them to the basolateral membrane, followed by exit via the heteromeric organic solute transporter OSTα/β (SLC51A/B) into the portal vein [170]. From there, conjugated bile acids travel back to the liver, where they are taken up by the hepatic sodium-dependent taurocholic transporter SLC10A1/NTCP [170]. Unconjugated bile acids can be taken up by OATP transporters (SLCO/SLC21 family).

The intestinal barrier poses a major challenge in the development of new strategies to improve oral drug availability. While most small molecule drugs administered orally are believed to be substrates of one or more uptake transporters expressed in the intestines [171,172], various approaches have been used to attempt to translocate non-transport substrates and poor substrates more efficiently across the brush border membrane. Bile acids and their derivatives were among the first molecules used to aid drug absorption in the intestines. Specifically, it was found that the conjugation of chemically modified cholic acid with peptides of different lengths resulted in the uptake of some of these peptides into bile [173,174], while competitively inhibiting the uptake of taurocholate. This was one of the first studies in this field when the molecular identity of the bile acid transporter in the ileum was still unknown. The latter was then successfully identified in 2003 as ASBT/SLC10A2 [175,176].

Subsequent studies have used the prodrug-based strategy to enhance either intestinal absorption or targeting to the liver. In the case of the antiviral agent ribavirin used to treat hepatitis C infections, the aim was to lessen off-target effects in erythrocytes that cause hemolytic anemia. To this end, ribavirin was conjugated to bile acids to target the liver bile acid transporter NTCP/SLC10A1 [176]. This strategy reduced the ribavirin concentration in erythrocytes 16.7-fold and the exposure of ribavirin in erythrocytes, plasma and kidneys 1.8-fold, while exposure in the liver was similar to that of ribavirin itself [177].

The antiviral agent valacyclovir was conjugated with chenodeoxycholate to improve oral bioavailability. The conjugate resulted in a more than 10-fold increase in intestinal uptake compared to the parent acyclovir in a cell line model [178]. In addition, the acyclovir molecule was recovered from the urine of rats after the administration of the conjugate, indicating that acyclovir was successfully cleaved and activated in the organism.

Another example is floxuridine, an antimetabolite used to treat metastatic liver disease. In order to enhance its hepatic uptake, it was conjugated with chenodeoxycholic acid using glutamic acid as a linker between the drug and the bile acid [179]. Two isomers were synthesized, and both were found to be substrates of NTCP/SLC10A1 [179]. The compounds showed stability in rat plasma but rapid release of the drug in rat liver. This suggests that glutamic acid is a promising linker for the conjugation of bile acids with liver-targeted drugs because the ester bond remains stable in plasma but is readily metabolized in the liver [179].

The conjugation of cytarabine, an anti-cancer agent that has poor oral bioavailability, with various bile acids has been explored as a way to improve intestinal absorption and liver targeting to optimize liver cancer treatment [180]. The ursodeoxycholic acid derivative of cytarabine showed prolonged half-life in vivo and increased oral bioavailability compared to cytarabine itself [180]. This confirms the benefit of the bile acid transporter-based prodrug strategy to enhance oral absorption.

Bile acid conjugation was also used to improve the oral bioavailability of heparin by the conjugation of deoxycholic acid with low molecular weight heparin (LMWH) [181]. The formulation was effective in vivo [182,183], and a competition study with free bile acid indicated that the uptake process was mediated by ASBT/SLC10A2 [183].

A similar strategy was later used with insulin by linking it to succinimido deoxycholate and succinimido bisdeoxycholyl-L-lysine [184]. The resulting conjugates retained high binding affinity to the insulin receptor and showed prolonged biological activity compared with normal insulin when administered intravenously to rats [184].

Another approach was taken by Lu and coworkers by linking paclitaxel to a PEG linker via a disulfide bond, which in turn was linked to cholic acid via an amide bond [185]. This targeted prodrug approach relies on elevated glutathione levels in tumor cells to reduce the disulfide bond and activate the drug [185,186,187,188]. The resulting formulation was resistant to acidic in vitro conditions mimicking those in the stomach, and the prodrug was able to enter MDA-MB-231 breast cancer cells, with uptake reduced by the addition of sodium taurocholate, indicating the involvement of ASBT/SLC10A2 [185]. In vivo studies in rats showed a higher plasma concentration of the prodrug than with paclitaxel administered alone [185]. For chemotherapeutics with limited solubility and permeability, this prodrug approach gives optimized oral delivery and tumor-specific release.

Early on, comparative molecular field analysis (CoMFA) suggested that substitution at positions 3, 7, 12 and 24 of bile acids lead to reasonable binding to the bile acid transporters [189]. Subsequent structure-activity studies confirmed that the C_2_-C_3_ positions of bile acids can successfully be conjugated without affecting their interactions with bile acid transporters [190]. In fact, C_3_ does not appear to specifically interact with ASBT/SLC10A2, and thus offers a preferred conjugation site [10,191], even though the C_3_ hydroxyl group seems to be essential for binding to IBABP [10]. In contrast, position 24 has frequently been used for conjugation, probably due to its easy chemical accessibility [191,192,193]. It is generally believed that the negative charge at the C_24_ position is not essential for transport, but significantly increases the affinity to bile acid transporters [191,193]. However, its modification might lead to a lower uptake rate [191].

Even though 3D-QSAR models successfully predict the interaction of small molecules with ASBT/SLC10A2 [194], the atomic-resolution structure of a bacterial homolog of ASBT from *Neisseria meningitidis* suggests that the cavity of ASBT is relatively small, and therefore it is questionable whether bile acid conjugates are actually transported [195]. On the other hand, it has also been claimed that the substrate-binding site of ASBT is much bigger than the size of bile acids, and that larger substrates can also be accommodated. For example, even a tetrameric form of deoxycholic acid shows high affinity for ASBT and improves the oral bioavailability of heparin upon conjugation [196]. Nevertheless, whether these large substrates actually enter the cell via the bile acid transporter or whether uptake occurs via another process such as endocytosis has not yet been clarified.

In addition to the classical prodrug strategy involving the direct chemical linking of small molecule drugs to bile acids, a nanocarrier strategy involving nanoparticles functionalized with bile acid molecules has also been developed. In particular, the decoration of various types of nanoparticles with bile acids has widely been used to enable the oral bioavailability of, for example, heparin and insulin.

Deoxycholic acid-conjugated chitosan particles were loaded with insulin for successful delivery into the portal vein [197,198,199]. Chitosan is a natural polysaccharide derived from marine crustaceans; chitosan-based nanomaterials have proven effective for advanced delivery approaches such as protein/peptide delivery, as they offer several advantages, including high encapsulation efficiency and favorable biocompatibility. Insulin-loaded deoxycholic acid-conjugated chitosan particles were shown to undergo ASBT/SLC10A2-mediated endocytosis, followed by sequestration to the basolateral membrane via IBABP and release at the basolateral membrane [197]. Another, different formulation based on the same idea has also been developed [200,201]. Polymer coating increases the stability of liposomes while enabling their conjugation with various small molecule ligands. Chitosan-coated and deoxycholic acid-modified liposomes have been successfully used to deliver insulin to rats in vivo [202], suggesting that the delivery of proteins/peptides via the bile acid uptake route is possible.

In another study, an attempt was made to deliver insulin by developing PEGylated polyhydroxybutyrate copolymeric nanoparticles conjugated with deoxycholic acid [203]. To avoid the release of insulin from the nanoparticles due to the harsh acidic and enzymatic milieu in the stomach, the nanoparticles were coated with a hydrophobic polymer, Eudragit S-100. While the encapsulation prolonged in vivo insulin release beyond 24 hours, deoxycholic acid ligation caused significantly higher intestinal uptake of the nanoparticles [203].

Heparin conjugated to nanomaterials has been explored in view of its expected versatility in the surface functionalization and embedding of biomolecules [204]. Nanocarriers were developed using heparin-taurocholic acid nanoparticles loaded with docetaxel [205,206]. The self-assembling formulation enabled effective oral absorption and anti-cancer activity in tumor-bearing mice and absorption could be blocked by the administration of taurocholic acid, confirming the involvement of the bile acid pathway.

In a further attempt to explore bile acid transporter-mediated delivery routes to improve oral administration of poorly water-soluble drugs, self-assembling hybrid nanoparticles of sodium-taurocholate and polyvinyl caprolactam-polyvinyl acetate-polyethylene glycol (Soluplus®, BASF Pharma, Germany) were prepared, and the calcium channel blocker felodipine was selected as a model drug [207]. The permeability of felodipine depended on the presence of taurocholate on the particles and was inhibited by excess sodium taurocholate or direct inhibition of ASBT/SLC10A2. A fluorescence approach was used to verify that the Soluplus nanoparticles were taken up intact by the ileum. These results confirm the potential use of this approach to enhance the oral bioavailability of poorly soluble drugs [207].

Taurocholic acid-modified nanostructured lipid carriers based on polyethylene glycol 100-monostearate have furthermore been developed for improving the oral delivery of the cancer preventing agent curcumin [32]. Taurocholine modification has also been used on nanoparticles to deliver siRNA of Akt2 for the treatment of colorectal cancer metastases in the liver [208].

Conjugation with glycocholic acid has also been shown to increase drug bioavailability. This has been shown for the 39-amino acid peptide exendin-4, a glucagon-like peptide-1 (GLP-1) receptor agonist and incretin mimetic used to treat type 2 diabetes. However, its therapeutic benefit is limited due to the frequent injections required. To address this issue, liposomes coated with glycocholic acid-conjugated chondroitin sulfate and loaded with exendin-4 were used to facilitate oral administration [209]. The efficiency of the nanoparticle formulation was similar to that of subcutaneous injection of exendin-4 in a rat model of type 2 diabetes. Interestingly, the site of absorption of the modified liposomes relocated from duodenum to ileum, most likely as a result of coating with bile acids [209].

To avoid problems with the bioavailability of the chemotherapeutic agent etoposide, a topoisomerase II inhibitor, this medication was embedded in a nanoemulsion based on low molecular weight methylcellulose, which also contains the ion pair of the anionic 1,2-didecanoyl-*sn*-glycero-3-phosphate, a lipid, and the cationic *N^α^*-deoxycholyl-L-lysyl-methylester, a derivative of deoxycholic acid [44]. This formulation showed improved cellular permeability in Caco-2/HT29-MTX-E12 cells and also higher oral bioavailability in in vivo studies in rats [44]. The inhibition of ASBT with actinomycin D and the heteromeric organic solute transporter OST α/SLC51A and OST β/SLC51B with clofazimine reduced permeability, indicating the involvement of bile acid transporters in this process [44].

It should be noted that the hydrophobic nature of certain bile acids, such as deoxycholic acid, causes the molecules to be preferentially buried in the liposomes and micelles, which could limit their efficacy by obstructing binding to bile acid transporters [8]. Similarly, while carriers such as bilosomes, which are liposome-like systems with bile acids present directly in the bilayer membrane, do show advantages for oral delivery, it is still unclear whether they directly interact with bile acid transporters [10].

### 3.4. Choline Transporters

Choline is an important precursor for phospholipid production in all cell types. In addition, it plays a special role in the brain for the synthesis of the neurotransmitter acetylcholine [210,211]. To meet the brain’s high demand for choline, and given the cationic charge of the choline molecule, it has been generally accepted that a dedicated choline transport system must be present at the BBB. However, its identity has long remained elusive. While the high-affinity Na^+^-dependent choline transporter CHT1/SLC5A7 was shown to be highly expressed in cholinergic nerve endings [212], it is not expressed in the brain capillary endothelial cells that form the BBB [213,214]. More recently, the choline transporter-like proteins CTL1/SLC44A1 and CTL2/SLC44A2 were shown to be expressed on the plasma membrane of human brain microvascular endothelial cells (hBMEC) as well as human brain cortical sections [214]. Upon the knockdown of CTL1/SLC44A1 by RNA interference in cultured rat astrocytes, the Na^+^-independent choline uptake activity vanished, indicating that CTL1 transports choline in a Na^+^-independent fashion [215]. Due to the presence of choline transporters at the BBB, they stand at the focus of drug-transporter interactions and serve as gateways for the delivery of therapeutic agents across the BBB. In addition to normal brain function, glioma cells have an increased demand for choline to synthesize phospholipids, which are essential for cell proliferation [8]. Therefore, targeting choline transporters could be beneficial both for delivering drugs into the CNS and for targeting glioma cells in the brain.

Even before the identification of the choline transporter at the BBB, pharmacophore models were proposed to study the chemical modifiability of choline while retaining affinity to its transporter [216,217]. Whereas earlier studies suggested that both the quaternary ammonium and the free hydroxyl groups are necessary for the recognition by the transporter [217], it was later found that bis-quaternary ammonium compounds can also inhibit transport [218]. Based on this, various linker lengths and types have been explored to develop high-affinity binders of the choline transporter at the BBB [219,220]. One of these compounds was shown to efficiently accumulate in the brain when linked to the BODIPY dye. Furthermore, a nanodelivery system based on dendrigraft poly-L-lysines (DLGs) decorated with the compound was able to successfully deliver plasmid DNA into the brain [220]. Interestingly, even though the uptake of conjugated nanoparticles was inhibited by excess choline, inhibition by filipine also suggested a non-specific adsorptive endocytosis mechanism of uptake [220].

Similar nanoparticles were later used to deliver a gadolinium chelate contrast enhancer for the localization of glioma by magnetic resonance imaging (MRI) [221], and also for the simultaneous delivery of doxorubicin and a vector carrying a gene encoding the hTRAIL (human tumor necrosis factor-related apoptosis-inducing ligand) protein [222]. Both applications showed superior brain penetration and activity of the formulations compared to non-conjugated controls. Similarly, a micellar preparation based on linking the above-mentioned choline derivative to a PEG segment conjugated with eight doxorubicin molecules was prepared and showed higher glioma accumulation compared to the same formulation without the choline derivative compound [223].

### 3.5. Vitamin Transporters

Vitamins are vital compounds that play a role as cofactors or precursors in a variety of fundamental physiological processes. Since vitamins are indispensable nutrients, there are numerous vitamin transporters in the intestines for their absorption. Several of these have been exploited as routes to enhance the oral absorption of drugs.

Ascorbic acid, or vitamin C, is an important cofactor in various enzymatic processes and typically acts as an electron donor. It also scavenges and neutralizes free radicals such as reactive oxygen species [224]. This antioxidant activity is especially important during the inflammatory reaction to protect immune cells [224]. The byproduct of the activity is the oxidized form of ascorbic acid, called dehydroascorbic acid (DHA) [224]. Different transport systems exist for these two forms. While L-ascorbic acid is taken up by the Na^+^-coupled vitamin C transporters SVCT1/SLC23A1 and SVCT2/SLC23A2, DHA can cross the membrane through facilitated diffusion with the help of GLUT/SLC2 transporters (see the section above about facilitative glucose transporters).

While SVCT1/SLC23A1 is expressed in epithelial tissues such as the small intestine and kidney and is responsible for regulating whole-body homeostasis of the vitamin, SVCT2/SLC23A2 is expressed more broadly. Importantly, SVCT2 is also highly expressed in epithelial cells of the choroid plexus [225,226], suggesting that it enables the transport of ascorbic acid into the brain [224,227]. This function is especially important because the blood levels of the oxidized form of vitamin C, DHA, which could serve as an alternative source of vitamin C supply to the brain, are negligible under normal physiological conditions [228]. Vitamin C taken up by SVCT2 in the epithelial choroid plexus cells was recently shown to exit the cells into the cerebrospinal fluid (CSF) via GLUT12/SLC2A12, a facilitative transporter that is highly expressed in the choroid plexus [229]. In further support of the concept that SVCT2 and GLUT12 provide vitamin C to the brain via the choroid plexus and the CSF, earlier autoradiographic studies confirmed that ^14^C-labeled ascorbic acid slowly accumulates in the central nervous system after intravenous injection and that radioactivity leaving the choroid plexus reaches the highest levels in the central nervous system about 6 days after intravenous injection in mice [230]. How exactly ascorbic acid enters the brain from the CSF, however, has not yet been clarified.

Since ascorbic acid can cross both the intestinal barrier and be delivered into the CSF via SVCT/GLUT ascorbic acid transporters, the conjugation of ascorbic acid to various compounds has been explored as a strategy for brain delivery.

Earlier studies to generate ascorbic acid transporter-specific ligands have shown that the C_5_ and C_6_ positions are modifiable without significantly affecting the interaction with the transporter [231,232,233]. Additionally, the oxidized forms of these derived compounds showed no interaction with GLUT1 and GLUT3, which transport DHA, confirming their specificity for SVCT transporters [231,232]. Some of these derivatives have been developed for medical imaging but have proven to be of limited use [234,235]. Prodrugs of nipecotic acid (an SLC6 GABA transporter uptake inhibitor), kynurenic acid (a neuroactive intermediate of L-tryptophan metabolism) and diclofenac acid (a nonsteroidal anti-inflammatory drug) conjugated with ascorbic acid have also been developed in order to improve their brain penetration [236,237,238]. The nipecotic acid derivative was also tested in a mouse model of epilepsy induced by pentylenetetrazole (a GABAA receptor antagonist) and was found to prolong the latency for the onset of tonic seizures, whereas the application of nipecotic acid itself had no such effect [237,238]. The γ-secretase inhibitor *N*-[*N*-(3,5-difluorophenylacetyl)-(*S*)-alanyl]-(*S*)-phenylglycine tert-butyl ester (DAPT), a potential therapeutic against Alzheimer’s disease, has also been chemically linked to ascorbic acid in order to improve its bioavailability in the CNS and to reduce potential off-target effects [239]. One of the developed compounds showed accumulation in the brain while retaining the inhibitory activity on γ-secretase [239]. A prodrug of the anti-inflammatory drug ibuprofen was also developed by conjugating ibuprofen with ascorbic acid to enhance its delivery to the brain via SVCT2, allowing it to be used for the treatment of CNS disorders such as Alzheimer’s disease [240]. The prodrug accumulated in the brain to a greater extent than ibuprofen and became activated in the brain, effectively releasing ibuprofen [240]. The uptake competed with the transport of free ascorbic acid, consistent with the involvement of SVCT2 in the uptake process [240].

To further exploit the potential of SVCT2 for improved drug delivery into the brain, liposomes and lipid-core polymeric micelles were developed as nanocarriers to target SVCT2. For this, the nanocarriers were decorated with ascorbate by modifying 1,2-distearoyl-*sn*-glycero-3-phosphoethanolamine-amino-PEG with ascorbic acid [227]. The nanocarriers showed enhanced targeting to SVCT2-expressing glioma cells based on the delivery of rhodamine into these cells, which could significantly be inhibited by the presence of free ascorbic acid in the medium, indicating SVCT2-mediated uptake [227]. In another study, poly(D,L-lactic-*co*-glycolic acid)-*block*-poly(ethylene glycol) (PLGA-*b*-PEG)-based nanoparticles were functionalized with ascorbic acid and loaded with galantamine, an acetylcholinesterase inhibitor used to treat Alzheimer’s disease [241]. Ascorbic acid conjugation enhanced uptake of the nanoparticles into SVCT2-expressing cells in in vitro studies. The functionalization also reduced the accumulation of galantamine in the liver, spleen, lungs and kidneys and improved the outcome in scopolamine-induced amnesic rats [241].

SMVT/SLC5A6 is a Na^+^-coupled vitamin transporter expressed in absorptive tissues such as the intestine, kidney and placenta [242,243]. It primarily plays a role in the intestinal absorption of the vitamins pantothenate and biotin, as well as lipoate, the enzyme cofactor that plays a key role in mitochondrial metabolism [243]. Even before the identification of this transporter, it was observed that the conjugation of biotin with various molecules enhances their cellular uptake [244,245,246]. In these studies, the cellular uptake of Tat (trans-activator of transcription) protein of the human immunodeficiency virus 1 (HIV-1) and its fragments were enhanced by biotin conjugation. The fragment proteins were developed to display Tat antagonistic activity, and in a later study, a retro-inverso derivative of this peptide was developed that exhibited high resistance to proteolysis in serum [247]. All of these Tat-derived biotinylated peptides were shown to use SMVT as an uptake route [247,248].

Similarly, a camptothecin topoisomerase inhibitor-PEG-biotin conjugate was developed, which showed enhanced cytotoxic activity compared with camptothecin alone [249]. Since the PEG and PEG-biotin fragments alone did not induce cell death, it was concluded that the improved efficiency of the conjugate was likely due to enhanced solubility, stability and SMVT-mediated uptake of camptothecin [249].

Prodrug derivatives of acyclovir conjugated with both various lipids and biotin were also developed, and the addition of both the hydrophobic moiety and biotin appeared to have an additive effect on increasing the cellular uptake of the compounds [250,251]. The effect of biotin conjugation could significantly be reduced by competition with biotin, indicating the involvement of SMVT in this process [250,251]. Computational docking of the biotinylated prodrug was also performed using a structural model of human SMVT, suggesting a possible mode of interaction of the generated compounds with the transporter [251].

Biotin has also been used to functionalize various nanocarriers in order to improve the oral bioavailability of biomolecules and drugs. Insulin encapsulated in biotinylated liposomes showed about twice the bioavailability of those in conventional liposomes [252]. The formulation exhibited a mild hypoglycemic effect that lasted longer than subcutaneous insulin injection [252]. Nanostructured lipid carriers functionalized with biotin were also developed for the intestinal absorption of oridonin, a natural compound with anti-inflammatory and anti-cancer effects, which otherwise exhibits low solubility and bioavailability [253]. However, the involvement of SMVT in the uptake of these formulations has not been tested.

Several types of cancer exhibit increased uptake of vitamins, indicating that vitamin transporters (or receptors) could be used for selective cancer targeting [254,255,256]. In one study, a hydrophobized polysaccharide, pullulan acetate, was used to generate self-assembling nanoparticles functionalized with biotin to enhance cancer cell targeting [257]. The biotin-conjugated nanoparticles showed increased uptake in cells of the HepG2 carcinoma cell line compared to unconjugated particles [257]. Biotin-coated nanodiamonds (i.e., carbon-based nanomaterials that provide large surface area for drug delivery) were developed and tested against streptavidin binding, but cell-based in vitro studies were not performed [258].

Another type of nanoparticles formed from poly(amido)amine (PAMAM) dendrimers conjugated to biotin and labeled with fluorescein isothiocyanate (FITC) were shown to be taken up by HeLa cells much more effectively than unmodified PAMAM [259,260]. However, the uptake of these nanoparticles was only partially mediated by SMVT, as it proceeded predominantly through nonspecific absorption that could not be inhibited by biotin [260,261]. While biotin conjugation did confer an advantage for FITC dye delivery, no increase in delivery was observed between biotinylated and non-biotinylated particles when the nanoparticles were loaded with cisplatin [261].

Biotin-conjugated polymeric micelles were also developed as delivery agents for doxorubicin. The corresponding study showed that biotin labeling enhanced both cellular uptake and drug efficacy when tested in the MCF-7 breast cancer cell line [262]. Similar results were reported for biotinylated cubosomes (i.e., liquid crystalline nano-structures formed from the cubic phase of lipids) carrying paclitaxel into HeLa adenocarcinoma cells [263] and for biotinylated polyurethane-urea nanoparticles loaded with a reporter gene-encoding vector and either sunitinib or phenoxodiol as anticancer agents [264]. However, none of these studies examined whether SMVT was involved in the uptake process.

In an interesting study showing that biotin targeting is likely receptor-mediated, rat erythrocytes were used as nanocarriers functionalized with *N*-hydroxysuccinimide ester of biotin [265]. Upon injection into rats, the modified erythrocytes accumulated predominantly in the liver and spleen, which was attributed to a clearance process of the biotinylated erythrocytes that depends on C3b receptors of the complement system present on liver and spleen macrophages, which then leads to opsonization and excretion by the liver and spleen [265]. Subsequently, methotrexate was encapsulated into the erythrocytes using the pre-swell dilution procedure and was shown to accumulate in the liver at higher levels one hour after application of the biotin-labeled erythrocytes compared to using unlabeled nanoparticles [265].

Vitamin B6 has also been used as a conjugate to enhance the uptake of nanoparticles into cancer cells [266], as increased vitamin B6 metabolism is associated with cancer risk, especially in lung cancer, and elevated expression of the vitamin B6-dependent enzyme serine hydroxymethyltransferase (SMHT) is associated with an increased requirement for DNA synthesis as part of the metabolic adaptation of cancer cells to support growth and proliferation [267,268]. Nanoparticles consisting of a poly(ester amine)-based gene delivery system were decorated with the active form of vitamin B6, pyridoxal 5’-phosphate. The decorated system exhibited higher transfection rates in lung cancer cells than normal lung cells, resulting in enhanced gene delivery within the rapidly proliferating cancer cells. The nanoparticles utilized an uptake mechanism with relatively high affinity, followed by an endocytic internalization mechanism. Moreover, the uptake of the nanoparticles could be inhibited by the vitamin B6 antagonist, 4’-deoxypyridoxine [266]. Whether uptake involves one of the known H^+^-coupled thiamine transporters SLC19A2 and SLC19A3, which mediate transmembrane translocation of the positively charged pyridoxine [269], or whether another, yet unidentified transporter is involved is still unclear.

The conjugation of folic acid has been used to improve the oral bioavailability of therapeutics, as a means to target cancer cells, and also to deliver drugs into the brain via the blood–cerebrospinal fluid barrier. The first folate transporter identified was the reduced folate carrier (RFC/SLC19A1), which shows high affinity for reduced folates most abundant in systemic circulation, such as 5-methyltetrahydrofolate. It is widely expressed and can mediate folate uptake from the bloodstream [270]. Intestinal absorption of dietary folic acid occurs in the duodenum and upper jejunum predominantly as a carrier-mediated process with a low-pH optimum [271]. The transporter responsible for uptake was identified as the H^+^-coupled folate transporter PCFT/SLC46A1 [272]. It enables folates to be absorbed across the brush-border membrane. PCFT is also expressed in the choroid plexus and is required for the transport of folates into the CSF. Loss of function of this transporter causes autosomal recessive hereditary folate malabsorption, a syndrome characterized by severe systemic and cerebral folate deficiency [272]. The folate receptor alpha (FRα) is expressed in the choroid plexus as well, and its loss of function results in an autosomal recessive disorder that solely leads to cerebral folate deficiency [272]. One theory to account for the requirement of both PCFT and FRα in the transepithelial flow of folate from blood to CSF is that folate binds from the blood side to the receptor at the basolateral membrane where PCFT is also expressed. This would be followed by internalization and the forming of a vesicle containing both receptor and PCFT, which would traffic to the apical membrane and be released into the CSF as an exosome from which folates are exported via PCFT [273]. An alternative pathway would comprise the PCFT-mediated export of folates from acidified endosomes within the intracellular compartment, followed by export into the CSF via the RFC/SLC19A1 reduced folate/organic phosphate antiporter [274].

Folate-functionalized PLGA nanoparticles have been successfully used to deliver an Hsp90 heat shock protein inhibitor to mouse Colon-26 epithelial-like and Raw 264.7 macrophage-like cells [275]. The formulation was also shown to be taken up by inflamed colon cells in a mouse model of ulcerative colitis and to attenuate both inflammation as well as colitis-associated cancer [275]. In contrast, similar nanoparticles without folic acid conjugation did not show therapeutic efficacy. Due to the expression of folate receptors on the inflamed colon cells, the cellular uptake was suggested to be a receptor-mediated process [275].

Interestingly, PCFT/SLC46A1 was shown to be upregulated in proximal intestinal epithelial cells of diabetic rats [276]. Based on this observation, folate-grafted chitosan nanoparticles were generated and loaded with insulin. The resulting nanoparticles could be taken up by Caco-2 cells highly expressing PCFT and transported through the Golgi pathway, while the uptake was attenuated by free folic acid. In contrast, in Caco-2 cells expressing lower amounts of PCFT, the nanoparticles were endocytosed but mainly degraded in lysosomes [276]. In vivo studies with diabetic rats also showed that the nanoparticles can successfully deliver insulin into the bloodstream reaching an oral bioavailability of 14.4% [276].

In humans, the receptor-mediated uptake pathway of folate can be mediated by three different folate receptors paralogs, α, β and γ [277]. While all three folate receptors are reported to be expressed in the small intestine only at negligible levels [271], folate receptor α is expressed in epithelial cells of the proximal tubules of the kidney and the choroid plexus, as well as in various cancers [277,278]. Due to this, folate-linked therapeutics have been developed for targeting cancer cells, which predominantly use the receptor-mediated pathway.

Folate-conjugated *N*-trimethyl-chitosan chloride (TMC) nanoparticles have been engineered for targeting tumor cells, which could be loaded with anti-cancer proteins [278]. In this study, the nanoparticles were loaded with FITC-BSA (bovine serum albumin), and folate functionalization showed a 3.7-fold increase in uptake compared to non-functionalized nanoparticles [278]. The dependence of the uptake on folate receptor expression was confirmed by competition with free folate in the buffer and by using the A549 folate receptor-deficient cell line [278].

Multi-walled carbon nanotubes were coated with chitosan that was previously functionalized with folic acid to generate a nanodelivery agent [279]. The nanoparticle was able to deliver a plasmid encoding green fluorescent protein (GFP) into HeLa and MCF-7 cancer cells, and the chitosan-folic acid coating improved the transfection efficiency 1.5-fold [279]. The uptake was suggested to be mediated by a folate receptor, but this has not been examined in detail.

Recently, a new route for the delivery of nanomedicine into the CNS was described using the folic acid transport pathway of the choroid plexus [280]. This was based on the observation that folate uptake in neuroepithelial cells in mouse embryos is dependent on the presence of the low-density lipoprotein (LDL) receptor-related protein 2 (LRP2) in the cellular membrane [281]. It was hypothesized that the direct interaction of the folic acid-bound soluble FRα with LRP2 triggers the endocytosis of the receptor complex and thus enables folic acid uptake [281]. To exploit this pathway, nanoparticles made of poly-(ethylene glycol)-*block*-poly(ε-caprolactone) (PEG-*b*-PCL) were surface-modified with the folic acid receptor α/folic acid complex (FRα-FA). The uptake of FRα-FA conjugated nanoparticles by human choroid plexus epithelial cells (HCPEpiCs) was determined in vitro using inverted optical fluorescence and confocal microscopy. FRα-modified nanoparticles were internalized by HCPEpiCs to a greater extent and the apparent permeability coefficient was significantly higher than that of their unmodified counterparts [280]. The biodistribution of unmodified and FRα-FA-modified nanoparticles following intravenous administration were compared in ICR albino mice and showed that conjugation of the FRα-FA complex to the nanoparticle surface promoted higher accumulation in the brain, highlighting the potential of FRα-FA-modified nanoparticles as a strategy for delivering molecules from the blood into the CNS. However, the mechanism of cellular uptake and transport of the nanoparticle across the choroid plexus and whether folic acid transporters play a role in this process remain unclear and require further investigation.

### 3.6. Oligopeptide Transporters (PEPT1/PEPT2)

The conjugation of amino acids and dipeptides to existing drugs has been a long-standing strategy to improve their oral bioavailability [282,283,284,285]. One of the first and most extensively studied prodrugs of this type is valacyclovir, the L-valine conjugate of the potent antiviral agent acyclovir [286]. The conjugation of acyclovir with amino acids such as L-valine significantly improved oral bioavailability as tested in rats [286] and also in human volunteers [287].

The intestinal transport system responsible for the increased permeability was identified as the oligopeptide transporter PEPT1/SLC15A1 [288,289]. It is highly expressed in the small intestines and is responsible for the absorption of dietary di- and tripeptides as well as a variety of peptide-like drugs such as aminocephalosporins, angiotensin-converting enzyme inhibitors, antiviral prodrugs and many others (see below) in a proton-coupled manner [288,290]. Its closest paralog with 50% sequence identity [291], PEPT2/SLC15A2, is expressed in the proximal tubules of the kidney, where it reabsorbs oligopeptides, as well as peptide-like drugs, including prodrugs [290]. PEPT2 is also expressed in adult rat brains by astrocytes, ependymal cells, subependymal cells and the epithelial cells of the choroid plexus [292]. Additionally, retinal Müller cells and peripheral satellite cells express this transporter [292]. Two further paralogs in humans, SLC15A3/PHT2 and SLC15A4/PHT1, are less well-characterized and are highly expressed in various immune cells, where they are thought to operate as histidine and peptide transporters [290,293]. SLC15A3 and SLC15A4 are preferentially expressed by cells within the lymphoid system, including dendritic cells, and are upregulated in response to toll-like receptor (TLR) stimulation [294,295]. Recent studies have shown that SLC15A4 contributes to the trafficking of TLRs and their ligands to endolysosomes, wherein recognition and signaling are initiated [296].

Since the identification of the PEPT1/SLC15A1 transporter as a promiscuous intestinal peptide uptake mechanism, a wealth of scientific literature has focused on the production of amino acid, dipeptide and tripeptide prodrugs to enhance intestinal absorption of drugs that are not readily absorbed through the oral route. Drug classes for which this strategy has been used include antiviral agents (acyclovir [286,287,289,297,298,299,300], gancyclovir [301], levovirin [302], oseltamivir [303,304], zanamivir [305], peramivir [306], lopinavir [307], cidofovir [308] and zidovudine [309]), chemotherapy medications (cytarabine [310], paclitaxel [311], floxuridine [312,313,314,315,316,317], gemcitabine [318] and melphalan [319]), anti-inflammatory agents (5-aminosalicylic acid [320], nabumetone [321] and ibuprofen [322]), natural products (oleanolic acid [323,324,325] and glucosamine [326]), L-DOPA and L-methyldopa [20,327,328,329,330], as well as tricin [331], pterostilbene [332], alendronate [333,334] and various other drugs [335]. An interesting strategy was the development of a dipeptide-like thiopeptide “carrier”, which is a small molecule binder of PEPT1 that was intended to be used as a general drug carrier [321]. This carrier was chemically conjugated to several different drugs, and many of the conjugates showed high-affinity binding to PEPT1 and the ability to permeate into cells and through a Caco-2 cellular monolayer [322,335].

Additionally, many studies have focused on exploring the selectivity of binding to PEPT1 to optimize interactions between prodrugs and the transporter. In a study using a dipeptide-conjugated azidothymidine library to screen the ability of dipeptides to compete with the known ligand cephalexin, certain dipeptides, such as Phe-Gly and Val-Ser, were found to be highly effective, in line with previous studies [336]. Peptides whose first amino acid is Ile or Ala have also shown binding to PEPT1, as has previously been found for certain prodrugs [336]. In addition, several other dipeptides (Arg-Ile, Ile-Ala, Leu-Ile, Phe-Ala, Phe-Lys, Pro-Ile, Ser-Pro, Ser-Glu, Thr-Ala and Val-Arg) have shown strong binding to PEPT1 [336], which could also be used in drug modifications to improve intestinal permeability. Other studies have also shown that L-valine and L-isoleucine conjugation often gives the most efficient cellular permeability and oral bioavailability [286,297,302,303,305,306,310,311,314,316,318,323,324,332]. In turn, D-amino acid-containing dipeptides appear to be less well-absorbed by the oligopeptide transport system, and their affinity is also lower [329].

Interestingly, as the structural basis of valacyclovir [337] and valganciclovir [338] binding to homologs of PEPT were experimentally elucidated, it was found that the two drugs bind in different orientations in these structures despite their very close chemical similarity. It was suggested that the different binding orientations of these prodrugs may depend on structural differences between the prokaryotic DtpA protein and mammalian PEPT proteins used in the above studies [339]. With the recent resolution of an outward-open state of rat PepT2, molecular docking suggested a binding mode for valacyclovir consistent with that of tripeptides [339]. Using the same protein structure, a very similar binding mode and set of interacting residues were proposed for valganciclovir, and these might correspond to an initial binding conformation [339]. Nevertheless, this mode of binding and the set of interacting residues do not completely overlap with the experimentally observed binding mode of valacyclovir to the inward-open structure of a bacterial homolog of hPEPT1, Pept_Sh_, which might represent a substrate conformation at a later point in the transport cycle [337].

Variation in binding modes has also been observed previously for di- and tripeptide substrates [340,341]. As for dipeptides with positively charged lysine and arginine sidechains, it was proposed that they could bind in a conformation similar to that of tripeptides [342]. The conserved position of the amino group in the above-mentioned binding modes of valacyclovir and valganciclovir [339] as well as di- and tripeptides [340,341] suggests that the *N*-terminus of substrate peptides is the primary binding site of the substrate, confirming previous findings and the importance of a free amino group in high-affinity substrates [343].

The chemical requirements for a PEPT1 substrate have been the subject of a number of studies. Early on, it was shown that PEPT1 substrates must have at least the two oppositely charged head groups separated by at least four methylene groups [344]. Based on structural information of rat PepT2, it was proposed that Glu622 and Arg57 are responsible for binding the *N*- and *C*-terminus, respectively, of the bound peptide substrate [339], corresponding to Glu595 and Arg27, respectively, in hPEPT1. The same cryo-EM structure has also confirmed that the ideal distance between these two binding points is about 6 Å, in line with previous suggestions [339,344]. In addition, it has been proposed that interaction with the first carbonyl group of the substrate through Asn192 (Asn171 in hPEPT1) and a series of tyrosine and tryptophan residues in the protein contributes to the promiscuity of the binding pocket by forming hydrophobic and polar subpockets as required [339,345]. The importance of a carbonyl moiety near the primary amino group of the substrate has also been shown to be important in previous studies of the structure–activity relationship [346].

It should be noted that in some instances, amino acids were conjugated with drugs via their amino groups while bearing a free carboxyl group, resulting in compounds that did not comply with the binding mechanism described above [306,331,332,347]. In one such case, although the uptake of such a prodrug competed with the PEPT1 model substrate glycylsarcosine (Gly-Sar), uptake was also inhibited by estrogen-3-sulfate, which is a typical substrate of the organic anion-transporter OATP2B1/SLCO2B1 that is also expressed in the Caco-2 cells used in the assay [332]. Consequently, anionic amino acid prodrugs intended to be substrates for PEPT1 may also utilize other cellular uptake pathways.

PEPT1 has also been targeted by nanoparticle formulations to enhance the intestinal absorption of drugs. One such example is the targeting of PEPT1 for the delivery of docetaxel by developing dipeptide-linked (L-valyl valine, L-valyl phenylalanine) PLGA nanoparticles [348]. The formulation was tested in HeLa cells stably transfected with hPEPT1, as well as in Caco-2 cells, and showed improved uptake compared to unmodified nanoparticles. In addition, in situ small intestinal perfusion as well as in vivo oral absorption experiments showed that the modified nanoparticles can elicit higher uptake of the drug and higher plasma half-life. Interestingly, the application of the formulation was observed to decrease the expression of PEPT1 at both the mRNA and protein levels [348].

PEPT1 was furthermore targeted using poly(lactic acid)-poly(ethylene glycol) (PLA-PEG) nanoparticles linked with valine, Gly-Sar, valyl-glycine and tyrosyl-valine to improve the oral delivery of acyclovir [349]. In vitro uptake of the functionalized nanoparticles competed with the PEPT1 model substrate Gly-Sar, indicating the involvement of the transporter in the uptake of the nanoparticles [349]. Encapsulation of acyclovir did not alter the rate of absorption, based on the assessment of the C_max_/AUC ratio, but increased the half-life and mean residence time (MRT) following oral administration in in vivo mouse studies [349].

### 3.7. Organic Cation Transporters (OCTNs)

OCTN2/SLC22A5 is a Na^+^-coupled L-carnitine transporter expressed in the small intestine and is responsible for the uptake of dietary L-carnitine [350,351,352] together with ATB^0,+^/SLC6A14 [353,354]. The carnitine uptake pathway has been used to improve oral absorption of various drugs. The prodrug strategy was used to synthesize a conjugate linking L-carnitine to gemcitabine through its carboxyl group. This formulation was able to increase the oral bioavailability of gemcitabine by up to 4.9-fold [355]. A potential prodrug of butyrate, butyrate-L-carnitine, has also been synthesized for the treatment of IBD because of its anti-inflammatory effect [126]. However, under pathophysiological conditions, OCTN2 was shown to be downregulated while SLC6A14 was expressed at higher levels, so the uptake of the prodrug likely proceeded via SLC6A14 [126] (see also the section on amino acid transporters). L-carnitine conjugation has also been used to increase the hydrophilicity and to prolong the pulmonary residence time of prednisolone, a drug often administered by inhalation for the treatment of bronchial asthma (BA) [356]. The non-tumorigenic human airway epithelial cell line BEAS-2B has been shown to express both OCTN2/SLC22A5 and OCTN1/SLC22A4 carnitine transporters [356,357]. Of the two prodrugs, the one retaining both the free choline and carboxyl groups of L-carnitine showed higher uptake in BEAS-2B cells, indicating that both charged groups are probably important for substrate recognition by carnitine transporters [356].

L-carnitine conjugation has also been used to guide the targeting of various nanoparticle formulations. PLGA nanoparticles conjugated with L-carnitine were engineered to be taken up by Caco-2 cells [358]. It was found that the optimal surface density of L-carnitine is about 10% to achieve the highest uptake efficacy [358]. It was also found that the uptake of nanoparticles competes with free L-carnitine in the buffer and that the uptake process is dependent on the presence of Na^+^, which is in line with the Na^+^-cotransporter mechanism of OCTN2 [358]. The uptake of the labeled nanoparticles was also inhibited by various endocytosis inhibitors such as indomethacin and chlorpromazine, indicating that endocytosis is involved as well [358]. Nanoparticles with 10% L-carnitine labeling also showed the most favorable pharmacokinetic parameters, including maximum plasma concentration (C_max_) and oral bioavailability compared to unlabeled PLGA nanoparticles [358].

OCTN2 is also highly expressed in brain capillary endothelial cells that make up the BBB [359,360,361,362]. L-carnitine was used as a carrier to deliver nipecotic acid, an anticonvulsant, through the BBB [363]. The conjugate prodrug was shown to be taken up and activated in vivo in the mouse brain and to prolong the latency to convulsions triggered by pentylenetetrazole [363].

Cells of the T98G glioblastoma multiforme cell line have also shown robust expression of OCTN2/SLC22A5 [45]. This cell line has been used as a model for targeting glioma and the BBB with PLGA nanoparticles conjugated with L-carnitine [45]. Various linker lengths were explored, with PEG-1000 showing the highest cellular uptake [45]. Uptake of L-carnitine conjugated nanoparticles followed the endocytic pathway, was dependent on Na^+^ and competed with free L-carnitine, in contrast to unlabeled particles [45]. L-carnitine conjugated PLGA nanoparticles loaded with paclitaxel were found to be 11-fold more enriched in mouse brains compared to non-conjugated nanoparticles. In addition, the cytotoxicity increased with the application of L-carnitine conjugated nanoparticles compared to non-conjugated nanoparticles and to Taxol, presumably due to enhanced cellular uptake, as shown by cytotoxicity assays in T98G cells. Finally, in vitro anti-glioma efficacy was evaluated in T98G spheroids, with paclitaxel-loaded L-carnitine conjugated nanoparticles showing enhanced toxicity [45].

Since SLC6A14 can also transport L-carnitine, albeit with a lower affinity than OCTN2/SLC22A5, similar nanoparticles have been shown to bind to and utilize both transporters in various cancer cell lines [364]. Such dual targeting has also been used to target LAT1 and SLC6A14 with a single nanoparticle formulation [129], as described in the section on amino acid transporters.

### 3.8. Organic Anion Transporters (OATPs)

Transporters of the SLCO/SLC21 family (also called the organic anion transporter family) typically exhibit a broad substrate range with a preference for negatively charged substrates. Because of these properties, they readily interact with a great variety of endogenous compounds and xenobiotics [365,366]. OATP1B1/SLCO1B1 and OATP1B3/SLCO1B3 are two members of the SLCO family that are considered to have liver-specific expression [367]. In certain cases, these transporters have been targeted to enhance liver-specific drug delivery in order to reduce off-target effects in other tissues.

The enzyme glucokinase is present in the liver, pancreas and brain, and converts glucose to glucose-6-phosphate for further metabolism, playing a central role in glucose homeostasis [11]. Its activators are among the potential next generation therapies for type 2 diabetes [368,369]. However, these compounds can cause hypoglycemia due to the overactivation of glucokinase in the pancreas, leading to the overproduction of insulin [11]. One of the strategies to reduce off-target effects was the development of hepatoselective glucokinase activators by targeting OATPs highly expressed in the liver [368,370]. With this in mind, a structure-activity relationship (SAR) study of *N*-heteroaryl acetamides revealed a hepatoselective glucokinase activator that, through the incorporation of a carboxyl group, enables hepatoselective uptake via OATPs while minimizing passive cellular uptake of the compound [368]. One of the resulting compounds proved to be a substrate for OATP1B1 and OATP1B3 and showed enhanced activity in isolated hepatocytes compared to systemic activators. In addition, it was able to normalize fasting plasma glucose levels in a diabetic rat model without causing hypoglycemia, in contrast to systemic activators [368]. The hepatoselective compound displayed a tissue distribution with a liver-to-pancreas ratio of 75-fold for rats and 58-fold for dogs [368].

Systemic stearoyl-CoA desaturase-1 (SCD1) is an enzyme that catalyzes the introduction of a cis-double bond between the C_9_ and C_10_ positions of various long chain saturated fatty acid-CoA esters, which has made it a promising target for the treatment of type 2 diabetes, dyslipidemia, obesity and metabolic diseases [371,372]. However, systemic inhibition of SCD1 causes side effects such as dry skin and hair loss, in addition to its intended effect of reducing de novo production of oleic acid in the liver [371]. Since SCD1 expression is highest in the liver, hepatoselective inhibitors were developed by exploiting transport through liver-specific OATPs [371,373]. The OATP-targeting homing moiety used was either a tetrazole acetic acid [371] or a nicotinic acid [373], both of which bear a free carboxyl group. Each of these compounds has been shown to be a substrate of OATP1B1 and OATP1B3 [371,373]. The tetrazole acetic acid derivative displayed a liver-to-plasma distribution ratio of >10-fold and a liver-to-skin ratio of >30-fold in various preclinical animals [371]. In turn, both compounds showed improved blood glucose clearance in an obese mouse model [371,373].

### 3.9. Monocarboxylate Transporters (MCTs)

MCT1/SLC16A1 is a monocarboxylate transporter that has been shown to be highly expressed on both the apical and basolateral membranes of enterocytes along the intestinal tract, particularly in the colon and rectum [374,375,376]. MCT1 is responsible for the uptake of short-chain fatty acids such as acetate, propionate and butyrate, which are important metabolites with anti-inflammatory effects for maintaining a healthy colon. They are produced by the bacterial fermentation of undigested fibers from complex carbohydrates by the intestinal microflora [377,378,379]. Due to its localization, MCT1 has gained focus as a possible entry pathway for therapeutics across the intestinal barrier.

XP13512 is a prodrug of gabapentin designed to be transported by intestinal nutrient transporters to enhance the oral bioavailability of gabapentin at therapeutic doses for treating neuropathic pain [380]. The acyloxy-alkyl carbamate modification changes the zwitterionic state of gabapentin due to conjugation at the amino group, while the free carboxyl group remains unmodified. The prodrug was subsequently found to act as a substrate for both MCT1 and SMVT transporters and to compete with their natural substrates [380]. In vitro transport studies in Caco-2 and MDCK cellular monolayers indicate that the prodrug is able to cross both cellular layers effectively [380]. This prodrug was later marketed as gabapentin enacarbil, an extended release formulation, and approved for use for the treatment of moderate to severe primary restless legs syndrome (RLS) [381,382,383].

In another study, 5-fluorouracil was conjugated with dicarboxylic acids to target MCT1 in order to enhance oral bioavailability [384]. The octanedioic acid ester derivative of 5-fluorouracil showed superior uptake properties compared to the unmodified drug in Caco-2 cells, as well as in monolayers [384]. The uptake could be inhibited by known inhibitors and substrates of MCT1 such as quercetin and butyrate, respectively, indicating the involvement of the transporter in the uptake process [384]. The prodrug also showed improved uptake rates according to in situ perfusion measurements as well as a 4.1-fold increase in oral bioavailability in rats [384].

A similar strategy was used for gemcitabine, an anti-cancer agent, to develop prodrugs with various linker lengths to improve oral bioavailability by targeting MCT1 in the intestine [385]. Out of five prodrugs, all showed uptake competing with butyric acid, indicating the involvement of MCT1, with prodrug 2 showing the highest affinity to MCT1 [385]. Interestingly, all prodrugs showed superior permeation properties in Caco-2 monolayers compared to previous gemcitabine prodrugs that utilized PEPT1 [318] or OCTN2 [355] transporters for uptake. Prodrug 2 with a 6-carbon linker also showed the highest oral bioavailability in rats [385].

In addition, MCT1/SLC16A1 was also found to be highly expressed in tumors [386]. For delivery into cancer cells, *O*-carboxymethyl chitosan nanoparticles were modified with acetic acid to help target them to MCT1-abundant membranes [40]. The resulting nanoparticles showed significantly higher uptake rates of carried docetaxel than unmodified liposomes in Caco-2 cells [40]. The conjugation of acetic acid significantly improved oral bioavailability, while drug uptake was competitively attenuated by free acetic acid, suggesting the involvement of MCT1 in the process [40]. The conjugated nanoparticles also showed significantly higher anti-tumor efficacy than did the unmodified liposome formulation of docetaxel [40]. The best uptake results were achieved with 45.14% conjugation with acetic acid, likely due to steric crowding effects that limit transporter binding, as has also been suggested in other reports [358,387,388].

Butyrate itself was also used to decorate PEG-based nanoparticles to enhance intestinal delivery [389]. The functionalized nanoparticles showed up to 2.84-fold increase in uptake in both E12 and Caco-2 cells compared to non-functionalized ones [389]. Free butyrate, as well as lactate and pravastatin were found to attenuate the endocytosis of the functionalized nanoparticles, indicating the involvement of MCT1 in the uptake process [389]. The decorated nanoparticles also showed a twofold increased uptake in an ex vivo ligated intestinal loop assay compared with normal nanoparticles [389]. The nanoparticle formulations were used to deliver insulin into rats, and it was found that butyrate conjugation enhances oral bioavailability of insulin to threefold higher levels compared with unconjugated nanoparticles, while the nanoparticle encapsulation prolonged its release [389].

In the BBB, MCT1/SLC16A1 is expressed at both the luminal and abluminal membranes of the brain capillary endothelial cells, and it plays an important role at the luminal membrane during the influx of lactate from the bloodstream into the brain [390,391]. Therefore, it has also been a target for the delivery of formulations to the brain. To this end, β-hydroxybutyrate conjugated solid lipid nanoparticles loaded with docetaxel were tested [392]. The study showed a significantly increased uptake in brain epithelial cells, which could be inhibited by β-hydroxybutyrate, indicating the involvement of MCT1, followed by an effective increase in docetaxel distribution in the brain [392].

Pluronic-85, a tri-block copolymer that self-assembles into micelles, has been used as a nanocarrier to deliver drugs through the intestinal and blood–brain barriers, as well as into tumors [393]. While this material has been at the focus of drug nanocarrier development due to its low toxicity and inhibition of several ABC transporters related to multi-drug resistance [394], it has also been shown to interact with OCTN2 and MCT1 in bovine brain microvascular endothelial cells (BBMEC), which often serve as a model system for the BBB [378,395]. However, whether MCT1 is involved in the uptake of the various formulations based on Pluronic-85 has not yet been studied in detail [378].

## 4. Conclusions and Outlook

As a convenient reference to the reader, we have summarized the transporters, their presence in various organs and on biological barriers, the assigned endogenous substrates, as well as the cell lines that have been used for testing for transporter-mediated uptake in the studies referenced in our review in Table 1. In general, the rational design of targeted delivery systems has mainly focused on specific organs with well-characterized physiology, such as the liver, and key biological barriers (e.g., blood–brain barrier and intestinal barrier). However, based on similar principles, it should also be possible to target other organs (e.g., heart, kidney, lung, pancreas, prostate, ovary or bone) and barriers (e.g., the blood–cerebrospinal fluid barrier and the blood–retinal barrier), even though only limited information on applications is available and development is often hampered by the lack of a detailed characterization and quantitation of the transporters available on these organs and barriers [11].

There are certain routes of drug administration that offer viable alternatives to oral delivery. For example, nasal delivery of therapeutics through the nasal cavity is attractive because olfactory neurons exposed in the nasal cavity provide direct access to the CNS, thus bypassing the BBB while also avoiding initial metabolism in the liver [514]. To date, many ABC and SLC drug transporters have been reported to be present in the nasal cavity [515,516]. While there is evidence that some of these transporters, such as the equilibrative nucleoside transporter ENT1/SLC29A1, can readily mediate the uptake of substrates such as [^18^F]fluorothymidine into the brain through the nasal route [517], these mechanisms appear to be underutilized in the development of new formulations. While certain nanoparticle formulations designed for nasal-to-brain delivery use functionalization through receptor ligands, there do not appear to be any reports of targeting transporters in the nasal cavity for drug delivery [518].

Similarly, the pulmonary route of administration through inhalation could be an interesting alternative because of the thin epithelial barrier and large surface area of the lungs [519], and lower expression of metabolic enzymes compared to the liver [519,520]. While drug transporters are expressed in mammalian airway epithelia, their exploitation through rational design to enhance delivery has remained scarce [521,522,523]. In addition, OCTN2 expressed in the trachea has been used for lung-specific drug targeting by developing an L-carnityl ester conjugate of prednisolone for pulmonary administration against bronchial asthma [356]. This novel prodrug also showed improved efficacy in an in vivo model of asthma [524]. However, other transporters in different cell types of the lung have, to our knowledge, not been used for organ-specific drug delivery.

Along the same lines, many other transporters besides those mentioned in this review could also be potential targets for tissue- and barrier-specific targeting of medications. Specifically, in the intestines, several known uptake systems are available, including the cholesterol transporter SLC65A2/NPC1L1 (Niemann–Pick C1-like 1) [525], the fatty acid transporter CD36 [526] or the long-chain fatty acid transporter proteins SLC27/FATP [527]. In terms of brain and CNS targeting, one vitamin that must enter the brain from the blood via the choroid plexus and CSF is riboflavin (vitamin B2). Riboflavin is an essential component of the brain and is not synthesized in mammalian tissues. Based on in vitro studies it was shown that there is a potent active transport system for riboflavin in the isolated rabbit choroid plexus [528], but its molecular identity appears to be unknown. The known riboflavin transporters belong to the SLC52 family, but these do not appear to provide active transport [529].

Recently, many SLC transporters have been shown to have very specific expression patterns, in contrast to non-transporter protein families [530]. Nevertheless, many SLC transporters remain relatively understudied, and more information about their localization and tissue expression patterns would be beneficial. With respect to transporter expression levels, it is important to note that interspecies differences in expression may limit the usefulness of preclinical animal models and that bridging the gap between these models and clinical trials is an important challenge to overcome [33].

In terms of the exploitation of individual transporters, the lack of specific binders to transporters with similar substrate specificity can be a bottleneck [75]. However, their discovery would greatly aid in the development of more advanced drug delivery systems. Once specific binders become available, dual or multiple targeting could also be an interesting approach, especially when targeting heterogenous cell populations such as in tumors [26,129].

Transporters can also modulate the efficacy of nanoparticle uptake without directly taking part in the process. In this context, it is important to note that systematic database analyses suggest that many more transporter-like proteins may be encoded in mammalian genomes than previously thought [5]. For example, MFSD2A/SLC59A1, a key transporter for docosahexaenoic acid uptake at the BBB, regulates caveolae-mediated transcytosis by modifying the lipid composition of the plasma membrane [531]. Transcytosis in BBB endothelial cells is exceedingly suppressed compared to peripheral endothelial cells [531]. Priming the BBB with MFSD2A inhibitors, followed by the application of transcytosis-employing nanoparticles carrying doxorubicin showed that doxorubicin was taken up 4.3-fold more effectively in this manner [531].

In addition to their advantages, many of the current transporter-targeted delivery formulations still have to overcome certain challenges. One such obvious barrier in targeting nutrient transporters is the occurrence of off-target effects due to the abundant expression of these transporters in healthy cells in a variety of tissues [29]. On the other hand, pathological conditions can also lead to changes in the expression pattern of certain transporters [33]. For example, GLUT1 expression in the BBB is decreased in patients at the early stage of Alzheimer’s disease [31,532]. It should also be considered that the application of transporter-targeted formulations themselves may affect the expression level of the corresponding transporter, including depletion from the cell surface [26,29,30,103,115,127,348,364]. Transporter-targeting formulations can also be expected to compete with the endogenous substrate of the transporter and thus block its uptake, which can lead to efficacy and safety concerns [33]. Overall, most transporter-targeted formulations are limited by their specificity, potential toxicity and absorption efficiency [10].

While the efficacy of absorption and a phenotypic readout are often used to evaluate the success of transporter-targeted delivery, the underlying mechanism of uptake is often not known in detail, especially with respect to the recycling of the targeted transporters [26,29]. In addition, transporter-targeted nanocarrier formulations often become trapped in lysosomes, which can cause their degradation and prevent their successful transcytosis to the basolateral membrane [10]. However, the specific mechanism of lysosomal escape is not known. Therefore, more comprehensive studies of the intracellular fate, including the mechanism of absorption and processing of nanocarrier formulations, are of great importance.

## Figures and Tables

**Table 1:** SLC transporters used for barrier and organ targeting. The gene symbols and protein names of transporters, their expression in organs and on biological barriers, endogenous substrates and cell lines referenced in this review that have been used to test for corresponding transporter-mediated uptake. Primary cells have not been listed, except in special cases.

Gene symbol/Protein Name	Tissue Expression	Barriers	Cell Lines	Substrates
SLC1A5/ASCT2	brain [396,397], adipocytes [398,399], testis [399], colon, small intestine, pancreas, stomach [400], kidney [400], activated T cells [401], lung, skeletal muscle [399]	likely on the basolateral membrane of epithelial cells and responsible for taking up nutrients from the blood [399]	9L [140,141], SF188 [140,141], A549 [145], A549/DPP [147], BT549 [146], DU145 [144], LNCaP [144], MDA-MB-231 [146], PC-3 [141]	L-Ala, L-Ser, L-Thr, L-Gln, L-Asn, L-Glu, L-Met, L-Leu, L-Gly, L-Val [399]
SLC2A1/GLUT1	erythrocytes [402], eye, retina [403], brain microvessels [403,404], choroid plexus [403], peripheral nerves [403], placenta [403], uterus [405], liver [406]	BBB, placenta, BCSFB, blood–aqueous barrier, blood–retina barrier, blood–nerve barrier [403]	MDA-MB-231 [59,66], Bel-7402 [38], BMEC [70], BV2 [35], Caco-2 [35], hCMEC/D3 [70], HEp-2 [37], HRPE [55], human erythrocyte membranes [56], MCF-7 [66], Neuro-2a [69], RG-2 [36,61], SH-SY5Y [62], U-87 [63]	D-glucose [402,407], DHA [408,409], glucosamine [410]
SLC2A4/GLUT4	adipose tissue, skeletal and cardiac muscle [411,412]		C1C12 (differentiated, with induction by insulin) [78]	D-glucose (2-deoxy-D-glucose) [413], glucosamine [410]
SLC2A5/GLUT5	small intestine [414], kidney [414], skeletal muscle [414], adipose tissue [414], testis, spermatozoa [415], brain [416]	intestinal [414,417], BBB [416]	MCF-7 [75,76,77], MCF10AneoT [77], MDA-MB-231 [75]	D-fructose [415,418]
SLC5A6/SMVT	heart, brain, placenta, lung, liver, skeletal muscle, kidney, pancreas [419], small intestine [243], brain microvessels [420,421]	intestinal [243], BBB [421], blood–retinal barrier [422]	Caco-2 [247,248,250,252], HeLa [259,263], HepG2 [257,264], HLCE-D36 [245,246], A2780/AD [249], A2780 [249], KB [259], MCF-7 [262], MDCK-MDR1 [250], MT2 [245], OVCAR-3 [260]	pantothenate, biotin [242], lipoate, iodide [423]
SLC6A14/ATB^0,+^	lung, trachea, salivary gland, mammary gland, stomach, pituitary gland, uterus, prostate, testis [117,424], small intestine [118], colon [117,118,424]	blood–air barrier [117,425,426], intestinal [118,119]	MCF-7 [125,129,364], A549 [127], BxPC-3 [129], Caco-2 [364], CCD841 [364], HCT116 [364], HepG2 [30], HT29 [364], LS174T [364]	neutral and cationic amino acids (L-Ile, L-Leu, L-Met, L-Val, L-Ala, L-Gly, L-Ser, L-Cys, L-Asn, L-Thr, L-Gln, L-Phe, L-Trp, L-Tyr, L-His, L-Lys, L-Arg), β-alanine, 3,4-DOPA [117]
SLC7A5/LAT1	brain [427,428], brain microvessels [79,80,86,429,430], retina [431], placenta [86,429,432,433], testis [86,429,434], ovary [434], colon [86,434], fibroblasts [435], bone marrow [429], lymph node [429], monocytes [436], macrophages [436], peripheral leukocytes [429], spleen [86], pancreas [434,437,438], thymus [429]	BBB [79,430], placenta [86,429], blood–retinal barrier [431,434], blood–testis barrier [434], blood–follicular barrier [434]	MCF-7 [97,108,110,439], ARPE-19 [83,92,97], C6 [103,113], GL261 [111,112], AsPC1 [99], Bel7402 [110], BxPC-3 [99], hCMEC/D3 [91], HeLa [116], MBEC4 [79], MDA-MB-231 [110], MIAPaCa-2 [99], PANC-1 [99], S180 [110]	large neutral L-amino acids (L-Leu, L-Ile, L-Phe, L-Met, L-Tyr, L-His, L-Trp, L-Val, D-Leu, D-Phe, D-Met, D-Leu) [86,429], T3, T4 [429]
SLC10A1/NTCP	liver [440], pancreas [441]		NTCP-transfected HEK293 [178,179], not expressed in HepG2 [442]	cholate, TC, GC, CDC, TCDC, GCDC, UDC, TUDC, LC, TLC, GLC, TLC, GLC, TDC [440], estrone-3-sulfate [443,444]
SLC10A2/ASBT	small intestine [445,446], colon [446], kidney [447], bile duct [448,449], gallbladder [450]	intestinal [445,446]	Caco-2 [44,183,185,189,196,197,201,202,206,208,451]	cholate, TDC, TC, DC, TCDC, CDC, TUDC, UDC, GDC, GCDC, GUDC [444]
SLC15A1/PEPT1	small intestine [288,452,453], colon [452], kidney [454], pancreas [455], bile duct [456], monocytes [457]	intestinal [288,452,453]	Caco-2 [289,297,298,299,300,301,302,303,304,305,306,307,310,313,314,315,316,318,319,320,321,322,324,329,332,334,335,346,347,348,349], AsPC-1 [315,316], Capan-2 [315,317], MDCK [307,331], A549 [325], MCF-7 [311]	di- and tripeptides [288,290,336]
SLC15A2/PEPT2	kidney [454], brain [292,458], choroid plexus, retina [292], peripheral nervous system [292,459], enteric nervous system [460], lung [461], mammary gland [462], heart [463], spleen [464]	BCSFB [292]	SKPT [301,346], MDCK [307,331]	di- and tripeptides [465,466]
SLC16A1/MCT1	bladder [467], brain [390,467,468], choroid plexus [469], stomach [467], intestine [467], small intestine [374,376,390,468], colon [374,376,467], erythrocytes [468], eye [468], retina [470], heart [390,467,468], kidney [390,467,468], liver [467,468], lung [467,468], mammary gland [471], muscle [467], skeletal muscle (red muscle) [390,468], ovary [467], placenta [471], spleen [467], testis [467,468], epididymis [468]	intestinal [374,376], BBB [390], BCSFB [469], blood–retinal barrier [470]	Caco-2 [40,380,384,385,389], 4T1 [40], bEnd [392], HT29-MTX-E12 [389], MDCK [380], PEAK^rapid^ [380], U373 [392]	butyrate [472], L-lactate, pyruvate, acetoacetate, D,L-3-hydroxybutyrate, α-oxoisohexanoate, α-oxoisovalerate [473], acetate, propionate [474,475]
SLC19A1/RFC	expressed in 68 human tissues, highest levels in placenta, liver and peripheral blood leukocytes, also in heart [476], small intestine, colon, kidney and choroid plexus [477]	placenta [476]	A549 [278], Colon-26 [275], HeLa [279], MCF-7 [279], Raw 264.7 [275], SKOV3 [278]	5-MTHF [478,479]
SLCO1B1/OATP1B1	liver [480]		not expressed in HepG2 [481], primary rat hepatocytes have been routinely used [368,371,373]	DHEAS, estradiol-17β-glucuronide, estrone-3-sulfate, PG E2, TXB_2_, LTC_4_, LTE_4_, T4, T3, TC [480], bilirubin, MGB, BGB, cholate [482], GC [483], TUDC, GUDC [484]
SLCO1B3/OATP1B3	liver [485]		HepG2 [180]	DHEAS, estradiol-17β-glucuronide [485], estrone-3-sulfate, LTC_4_, T4, T3 [483], TC, GC [483,486], bilirubin [487], MGB [482], glutathione, cholate, TDC, TCDC [486], TUDC, GUDC [484]
SLC22A5/OCTN2	brain [488,489,490,491,492,493], brain capillary endothelial cells [359], spinal cord [490], retina [494], heart [489,490,491,492,493], salivary gland [492], small intestine [351,488,490,491,492], colon [488], kidney [488,489,490,491,492,493], liver [488,489,490,492], pancreas [489,490], trachea [490], lung [489,490,492], uterus [490], placenta [488,489,490,491,493,495], prostate [490], testis [488], skeletal muscle [488,489,490,491], striated muscle [492], adrenal gland [490,492], mammary gland [496], thymus [490], thyroid [490]	intestinal [351], BBB [359], blood–retinal barrier [494]	Caco-2 [355,358,364], BEAS-2B [356,357], RBE4 [361,362], BxPC-3 [355], CCD841 [364], hCEMC/D3 [45], HCT116 [364], HT29 [364], LS174T [364], MDA-MB-231 [364], MCF-7 [364], T98G [45]	L-carnitine, betaine [497]
SLC23A1/SVCT1	kidney, liver, small intestine, colon, ovary, prostate, pancreas [225,498], lung [499], skin [500]	intestinal [501]		L-ascorbic acid [498]
SLC23A2/SVCT2	brain, spleen, prostate, testis, ovary, placenta, peripheral blood leukocytes [498], retina, small intestine, epididymis, brain, choroid plexus, pancreas, adrenal gland, gastric glands, spleen, thymus, testis [225], lung [225,499], skin [500]	BCSFB [225,226], blood–retinal barrier [225], intestinal [225,498]	HRPE [236,237,238], NIH/3T3 [227,241], CRL-1497 [232], C6 [227], F98 [227]	L-ascorbic acid [498]
SLC44A1/CTL1	spinal cord, brain [502,503], lung [502,503], colon [502], peripheral blood monocytes and neutrophils, fibroblasts [504], brain microvessels [214], skeletal muscle, heart, testis [505], placenta, kidney, liver, small intestine, pancreas, spleen, ovary [503], mitochondria [214,506]	BBB [214], intestinal [502]	U-87 MG [221,222], brain capillary endothelial cells (BCECs) [220]	choline [502,505,507], ethanolamine [508]
SLC44A2/CTL2	brain [214,509], inner ear [509,510], stomach [511], intestine [511], colon [509], kidney [509,511], heart [509,511], lung [509,511], muscle [509,511], tongue [509,511], liver [509], spleen [509], testis [511], mitochondria [214,506]	BBB [214]	U-87 MG [221,222], brain capillary endothelial cells (BCECs) [220]	choline [357,508,509], ethanolamine [508]
SLC46A1/PCFT	kidney, liver, placenta, small intestine, colon, spleen, brain, testis, skin, stomach [271,512], choroid plexus [513]	intestinal [271,512], BCSFB [513]	A549 [278], Caco-2 [276], Colon-26 [275], HeLa [279], MCF-7 [279], Raw 264.7 [275], SKOV3 [278]	folic acid, 5-MTHF [271]

Abbreviations: 3,4-DOPA, 3,4-dihydroxyphenylalanine; 5-MTHF, 5-methyltetrahydrofolate; BGB, bisglucuronosyl bilirubin; CDC, chenodeoxycholate; DC, deoxycholate; DHA, dehydroascorbic acid; DHEAS, dehydroepiandrosterone-3-sulfate; GC, glycocholate; GCDC, glycochenodeoxycholate; GDC, glychodeoxycholate; GLC, glycolithocholate; GUDC, glycoursodeoxycholate; LC, lithocholate; LTC_4_, leukotriene C_4_; LTE_4_, leukotriene E_4_; MGB, monoglucuronosyl bilirubin; PG E_2_, prostaglandin E_2_; T3, triiodothyronine; T4, thyroxine; TC, taurocholate; TCDC, taurochenodeoxycholate; TDC, taurodeoxycholate; TLC, taurolithocholate; TUDC, tauroursodeoxycholate; TXB_2_, thromboxane B_2_; UDC, ursodeoxycholate.

## Data Availability

Not applicable.
